# International society of sports nutrition position stand: energy drinks and energy shots

**DOI:** 10.1080/15502783.2023.2171314

**Published:** 2023-03-02

**Authors:** Andrew R. Jagim, Patrick S. Harty, Grant M. Tinsley, Chad M. Kerksick, Adam M. Gonzalez, Richard B. Kreider, Shawn M Arent, Ralf Jager, Abbie E. Smith-Ryan, Jeffrey R. Stout, Bill I. Campbell, Trisha VanDusseldorp, Jose Antonio

**Affiliations:** a Sports Medicine, Mayo Clinic Health System, La Crosse, WI, USA; b Exercise & Sport Science, University of Wisconsin – La Crosse, La Crosse, WI, USA; c Exercise & Performance Nutrition Laboratory, Lindenwood University, St. Charles, MO, USA; d Energy Balance and Body Composition Laboratory, Department of Kinesiology & Sport Management, Texas Tech University, Lubbock, TX, USA; e Department of Allied Health and Kinesiology, Hofstra University, Hempstead, NY, USA; f Exercise & Sport Nutrition Lab, Department of Kinesiology and Sport Management, Texas A&M University, College Station, TX, USA; g Department of Exercise Science, Arnold School of Public Health, University of South Carolina, Columbia, SC, USA; h Increnovo LLC, Whitefish Bay, WI, USA; i Applied Physiology Laboratory, Department of Exercise & Sport Science, University of North Carolina, Chapel Hill, NC, USA; j School of Kinesiology and Rehabilitation Science, University of Central Florida, Orlando, FL, USA; k Performance & Physique Enhancement Laboratory, University of South Florida, Tampa, FL, USA; l Bonafede Health, LLC, JDS Therapeutics, Harrison, NY, USA; m Department of Health and Exercise Sciences, Jacksonville University, Jacksonville, FL, USA; n Department of Health and Human Performance, Nova Southeastern University, Davie, FL, USA

## Abstract

Position Statement: The *International Society of Sports Nutrition* (ISSN) bases the following position stand on a critical analysis of the literature regarding the effects of energy drink (ED) or energy shot (ES) consumption on acute exercise performance, metabolism, and cognition, along with synergistic exercise-related performance outcomes and training adaptations. The following 13 points constitute the consensus of the Society and have been approved by the Research Committee of the Society: Energy drinks (ED) commonly contain caffeine, taurine, ginseng, guarana, carnitine, choline, B vitamins (vitamins B1, B2, B3, B5, B6, B9, and B12), vitamin C, vitamin A (beta carotene), vitamin D, electrolytes (sodium, potassium, magnesium, and calcium), sugars (nutritive and non-nutritive sweeteners), tyrosine, and L-theanine, with prevalence for each ingredient ranging from 1.3 to 100%. Energy drinks can enhance acute aerobic exercise performance, largely influenced by the amount of caffeine (> 200 mg or >3 mg∙kg bodyweight [BW^−1^]) in the beverage. Although ED and ES contain several nutrients that are purported to affect mental and/or physical performance, the primary ergogenic nutrients in most ED and ES based on scientific evidence appear to be caffeine and/or the carbohydrate provision. The ergogenic value of caffeine on mental and physical performance has been well-established, but the potential additive benefits of other nutrients contained in ED and ES remains to be determined. Consuming ED and ES 10-60 minutes before exercise can improve mental focus, alertness, anaerobic performance, and/or endurance performance with doses >3 mg∙kg BW^−1^. Consuming ED and ES containing at least 3 mg∙kg BW^−1^ caffeine is most likely to benefit maximal lower-body power production. Consuming ED and ES can improve endurance, repeat sprint performance, and sport-specific tasks in the context of team sports. Many ED and ES contain numerous ingredients that either have not been studied or evaluated in combination with other nutrients contained in the ED or ES. For this reason, these products need to be studied to demonstrate efficacy of single- and multi-nutrient formulations for physical and cognitive performance as well as for safety. Limited evidence is available to suggest that consumption of low-calorie ED and ES during training and/or weight loss trials may provide ergogenic benefit and/or promote additional weight control, potentially through enhanced training capacity. However, ingestion of higher calorie ED may promote weight gain if the energy intake from consumption of ED is not carefully considered as part of the total daily energy intake. Individuals should consider the impact of regular coingestion of high glycemic index carbohydrates from ED and ES on metabolic health, blood glucose, and insulin levels. Adolescents (aged 12 through 18) should exercise caution and seek parental guidance when considering the consumption of ED and ES, particularly in excessive amounts (e.g. > 400 mg), as limited evidence is available regarding the safety of these products among this population. Additionally, ED and ES are not recommended for children (aged 2-12), those who are pregnant, trying to become pregnant, or breastfeeding and those who are sensitive to caffeine. Diabetics and individuals with preexisting cardiovascular, metabolic, hepatorenal, and/or neurologic disease who are taking medications that may be affected by high glycemic load foods, caffeine, and/or other stimulants should exercise caution and consult with their physician prior to consuming ED. The decision to consume ED or ES should be based upon the beverage’s content of carbohydrate, caffeine, and other nutrients and a thorough understanding of the potential side effects. Indiscriminate use of ED or ES, especially if multiple servings per day are consumed or when consumed with other caffeinated beverages and/or foods, may lead to adverse effects. The purpose of this review is to provide an update to the position stand of the *International Society of Sports Nutrition* (ISSN) integrating current literature on ED and ES in exercise, sport, and medicine. The effects of consuming these beverages on acute exercise performance, metabolism, markers of clinical health, and cognition are addressed, as well as more chronic effects when evaluating ED/ES use with exercise-related training adaptions.

## Introduction

The International Society of Sports Nutrition (ISSN) first published a position stand on energy drinks (ED) in 2013 [1]; however, since the publication of the initial position stand, an increased number of publications in the literature have focused on performance and cognitive effects, in addition to more data examining the safety of these beverages. Additionally, the ingredient profiles of ED have changed over the past decade, which may influence the ergogenic potential as well as their safety profile. Therefore, the current position stand serves as an updated review and consensus of the currently available literature focused on energy drinks and energy shots on behalf of the ISSN.

Energy drinks are pre-packed, ready-to-drink functional beverages, while energy shots (ES) are a similar more concentrated ready-to-drink beverage sold in 2-5 fl oz. volumes [2]. Historically, they were first introduced to the market in the United States (US) in the late 1990’s and have since become one of the biggest selling functional beverages in the industry with annual sales projected to surpass $21 billion by the year 2026 [3]. Globally, energy drinks have been in the marketplace since at least 1962 when Krating Daeeng (precursor to today’s Red Bull®) was first unveiled and sold in Thailand [4]. Energy drinks are frequently marketed towards active younger adults and adolescent populations, which also represent the majority of the consumer base (aged 20–39 years) [5]. For example, a 2013 study [6] which included survey respondents from 16 different European countries found a lifetime prevalence of ED use among adolescents (10-18 years. of age) to be ~68%, compared to 30% among adults. A sub-analysis indicated that of regular ED consumers, 12% were classified as ‘high chronic users’, which established an average consumption rate of seven liters per month (i.e. 14, 16 oz. beverages). Similarly, reports from Canada [7] and Germany [8], have found comparable prevalence rates of ED consumption among adolescents to be 62% and 61%, respectively. A recent study reported an increase in the prevalence of ED consumption from 2003 to 2016 within the US among adolescents (aged 12-19 years [0.2% to 1.4%]), young (aged 20-39 years [0.5% to 5.5%]) and middle-aged (aged 40-59 years [0.0% to 1.2%]) adults. These findings indicate that ED are already popular among adolescent and young adult populations. Energy shots are often manufactured and sold in 2-5 fl oz. ready-to-drink containers and as reported by Jagim et al. [2], have similar combinations of ingredients as observed in ED, albeit in different amounts due to obvious differences in the volume in which they are consumed (ES: 2-5 fluid ounces vs. ED: 12-16 fluid ounces). In terms of labeling, full ingredient disclosure (type and amount) is not required. However, it is our view that companies who sell EDs or ES as food beverages and/or dietary supplements should fully disclose the ingredients and amounts contained in the beverage so consumers can make an informed decision to limit inadvertent overconsumption of nutrients if necessary.

Recent research indicates the most prevalent ingredients found in ED are caffeine, B-vitamins (i.e. Vitamin B6, Vitamin B3, Vitamin B12, and Vitamin B5), taurine, ginseng, and carnitine [2], which explains them commonly being marketed to achieve increased alertness, focus, and energy [2]. From a regulatory perspective, the Nutrition Facts Panel on food labels is not required to always list caffeine since it is not a nutrient. However, if caffeine is added to a food, it must then be listed. Consequently, the caffeine content and other non-nutrients found in ED are not always fully disclosed on the label. Some ED are sold as food beverages (i.e. sodas and sports drinks) while others are sold as dietary supplements. This not only affects labeling claims but also labeling at point of purchase (food aisles or pharmacy/nutrition/dietary supplement sections of a store).

The purpose of this position stand was to: (1) provide a brief review of the key ingredients found in ED and ES; (2) summarize the scientific literature on the acute ergogenic effects of ED and ES; (3) evaluate the effects of continued use and long-term adaptations associated with ED and ES, along with safety considerations; (4) provide recommendations for the use of ED and ES.

## 2. Methods

ISSN position stands are invited papers on topics that the ISSN Research Committee identifies as being of interest to the readers of the *Journal of the International Society of Sports Nutrition* as well as any individuals that express an interest in that subject. Editors and/or the Research Committee identify a lead author or team of authors to perform a comprehensive literature review. The draft is then sent to leading scholars for a detailed review and approval. The paper is then revised as a consensus statement and reviewed and approved by the Research Committee and editors as the official position of the ISSN. The authors utilized a scoping review approach and included articles with a primary emphasis on physical and cognitive performance outcomes following consumption of ED or ES beverages conducted on human research participants. Importantly, the reader should understand that investigations which employed energy supplements in capsules or powdered formulations (i.e. caffeine, green tea, multi-ingredient pre-workout supplements, thermogenic [‘fat-burning’] products, etc.) were excluded from this position stand.

## 3. Brief review of key ingredients

A recent publication detailed the ingredient profiles of 75 top selling EDs (last accessed on 7 September 2021) and ES [2], from which the prevalence and quantities of ingredients included in these products are displayed in Tables 1 and 2. Common ingredients, each of which are described below, included caffeine, taurine, ginseng, guarana (as a source of natural caffeine), carnitine, choline, B vitamins (vitamins B1, B2, B3, B5, B6, B9, and B12), vitamin C, vitamin A (beta carotene), vitamin D, electrolytes (sodium, potassium, magnesium, and calcium), sugars, sweeteners (nutritive or non-nutritive), tyrosine, and L-theanine. The prevalence of each of these ingredients in ED and ES ranged from 1.3 to 100%. ES have similar combinations of ingredients as observed in ED, albeit in different amounts or concentrations due to differences in the volume in which they are consumed (ES: 2-5 fl. oz. vs. ED: 12-16 fl. oz.).

**Table 1.** Prevalence and quantities of ingredients (disclosed and undisclosed) included in bestselling energy drinks and energy shots (n = 75). *Reproduced from Jagim et al. 2021* [2], *which is licensed under an open access Creative Commons CC BY 4.0 license.*

Ingredient	Overall Prevalence (%)	Prevalence in Undisclosed Quantity (%)	Prevalence in Listed Quantity (%)	Mean ± SD Listed Quantity
Caffeine (mg)	100	0	100	174 ± 81
Taurine	37.3	37.3	0	N/A
Ginseng	30.7	30.7	0	N/A
Guarana	25.3	25.3	0	N/A
Carnitine	16.0	16.0	0	N/A
Choline (mg)	2.7	0	2.7	267 ± 330
Vitamin B1 (% of DV)	1.3	0	1.3	25.0
Vitamin B2 (% of DV)	8.0	0	8.0	133 ± 81
Vitamin B5 (% of DV)	37.3	0	37.3	114 ± 77
Vitamin B6 (% of DV)	72.0	0	72	367 ± 648
Folate (mcg)	6.7	0	6.7	258 ± 194
Vitamin B12 (% of DV)	66.7	0	66.7	5,245 ± 10,475
Vitamin C (% of DV)	22.7	0	22.7	59.8 ± 48.7
Vitamin D (% of DV)	2.7	0	2.7	35.0 ± 21.2
Vitamin A (% of DV)	6.7	0	6.7	78.6 ± 86.8
Sodium (mg)	70.7	0	70.7	120 ± 118
Potassium (mg)	34.7	0	34.7	148 ± 197
Magnesium (mg)	12.0	0	12.0	25.4 ± 23.4
Niacin (% of DV)	66.7	0	66.7	121 ± 70
Calcium (mg)	17.3	0	17.3	128 ± 175
Sugars (g)	45.3	0	45.3	19.9 ± 18.2
Tyrosine	22.7	22.7	0	N/A
L-Theanine	17.3	17.3	0	N/A

**Table 2.** Product class-specific prevalence and quantities of select ingredients included in bestselling energy drinks and energy shots (n = 75). *Reproduced from Jagim et al. 2021* [2], *which is licensed under an open access Creative Commons CC BY 4.0 license.*

	Energy Drinks (*n* = 55)		Energy Shots (*n* = 20)	
Ingredient	Prevalence in Product Class (% out of 55)	Mean ± SD Listed Quantity	Prevalence in Product Class (% out of 20)	Mean ± SD Listed Quantity
Caffeine (mg)	100	159 ± 74	100	217 ± 87
Vitamin B6 (% of DV)	74.5	165 ± 199	65.0	1,004 ± 1,069
Sodium (mg)	76.4	143.1 ± 120.7	55.0	30.5 ± 32.6
Niacin (% of DV)	70.9	115 ± 70	55.0	143 ± 70
Vitamin B12 (% of DV)	63.6	1,151 ± 4,020	75.0	14,796 ± 14,323
Sugars (g)	52.7	22.0 ± 18.9	25.0	7.6 ± 4.6
Vitamin B5 (% of DV)	47.3	111 ± 78	10.0	150 ± 71
Vitamin C (% of DV)	25.5	63.6 ± 49.2	15.0	41.7 ± 51.4
Calcium (mg)	18.2	160 ± 189	15.0	19.0 ± 17.8
Magnesium (mg)	12.7	26.1 ± 24.6	10.0	22.8 ± 27.2

Ingredient prevalence represents the proportion of each ingredient per product class.

In the U.S., conventional foods and beverages containing added caffeine must list caffeine as an ingredient, however, the quantity must does not have to be disclosed. In addition, foods and beverages containing naturally occurring caffeine do not need to label that the product contains caffeine [9]. Consequently, the caffeine content and other non-nutrients found in ED are not always fully disclosed on the label. However, ES are classified as dietary supplements and therefore are subject to different regulatory standards compared to ED. In terms of labeling, full ingredient disclosure (type and amount) is not required; however, compliance with all dietary supplement labeling regulations is required.

Importantly, the frequent presence of the ingredients discussed below in ED and ES does not imply that these ingredients are ergogenic. Furthermore, even when potentially ergogenic ingredients are present, doses may be lower than that which is demonstrated to be ergogenic – or conversely, higher than required. In many cases, there may be insufficient research to establish ergogenicity or a minimum effective dose of individual ingredients. An additional complication is that precise quantities of several ingredients below are not typically specified on the labeling of popular ED and ES products. Nonetheless, these brief summaries describe the prevalence, dose (when available), and potential for ergogenicity of ingredients commonly observed in ED and ES products.

### Caffeine

Caffeine is a naturally occurring methylxanthine alkaloid that is structurally related to the molecule adenosine [10]. Caffeine binds to adenosine receptors in the central nervous system, thereby inhibiting adenosine binding and exerting psychotropic effects [10,11]. This action promotes reduced fatigue sensation, increased subjective energy and alertness, and performance benefits across the exercise spectrum [12,13]. Caffeine is considered a primary ingredient in ED and ES, with 100% of the 75 bestselling products containing this ingredient [2], with an average dose (mean ± SD) of 174 ± 81 mg∙serving^−1^ (Tables 1-2). In comparison, the average cup of brewed coffee contains 80-150 mg of caffeine (per 8-12 oz serving), with some commercial coffees containing up to 480 mg (per 20 oz. serving) [14] as summarized in Table 3. Recently, the *International Society of Sports Nutrition* published an updated position stand on caffeine and exercise performance [13], with the following conclusions:

1. Supplementation with caffeine has been shown to acutely enhance various aspects of exercise performance in many but not all studies. Small to moderate benefits of caffeine use include, but are not limited to muscular endurance, movement velocity, and muscular strength, sprinting, jumping, and throwing performance, as well as a wide range of aerobic and anaerobic sport-specific actions.
2. Aerobic endurance appears to be the form of exercise with the most consistent moderate-to-large benefits from caffeine use, although the magnitude of its effects differs between individuals.
3. Caffeine has consistently been shown to improve exercise performance when consumed in doses of 3-6 mg∙kg BW^−1^. Minimal effective doses of caffeine currently remain unclear, but they may be as low as 2 mg∙kg BW^−1^. Very high doses of caffeine (e.g. 9 mg∙kg^−1^) are associated with a high incidence of side-effects and do not seem to be required to elicit an ergogenic effect.
4. The most commonly used timing of caffeine supplementation is 60 min pre-exercise. Optimal timing of caffeine ingestion likely depends on the source of caffeine. For example, as compared to caffeine capsules, caffeine chewing gums may require a shorter waiting time from consumption to the start of the exercise session.
5. Caffeine appears to improve physical performance in both trained and untrained individuals.
6. Inter-individual differences in sport and exercise performance as well as adverse effects on sleep or feelings of anxiety following caffeine ingestion may be attributed to genetic variation associated with caffeine metabolism, and physical and psychological response. Other factors such as habitual caffeine intake also may play a role in between-individual response variation.
7. Caffeine has been shown to be ergogenic for cognitive function, including attention and vigilance, in most individuals.
8. Caffeine may improve cognitive and physical performance in some individuals under conditions of sleep deprivation.
9. The use of caffeine in conjunction with endurance exercise in the heat and at altitude is well supported when dosages range from 3 to 6 mg∙kg^−1^ and 4-6 mg∙kg^−1^, respectively.
10. Alternative sources of caffeine such as caffeinated chewing gum, mouth rinses, energy gels, and chews have been shown to improve performance, primarily in aerobic exercise.
11. Energy drinks and pre-workout supplements containing caffeine have been demonstrated to enhance both anaerobic and aerobic performance.

**Table 3.** Caffeine content per serving of caffeine containing products.

Beverage	Caffeine (mg)
Energy Drinks [2]	160
RedBull© (12 oz.)	111
BANG© (16 oz.)	300
Coffee (8 oz.)	90
Commercial Coffee (20 oz.)	410
Green Tea (16.9 oz.)	94
Soda (12 oz.)	35
Pre-Workout Supplements [1]	254
Caffeine Capsule	200

Oz = fluid ounces.

In healthy adults, the Food and Drug Administration (FDA) within the United States suggests that a caffeine intake of 400 mg∙d^−1^ is not commonly associated with adverse effects, while the American Medical Association recommends a limit of 500 mg∙d^−1^ for adults and 100 mg∙d^−1^ for adolescents [11,15,16]. When considering body size, previous research recommends keeping caffeine intake within 3 – 6 mg∙kg^−1^, as this is the level of intake that is ergogenic and found to be well-tolerated, regardless of size and age [13]. Given this, it is important to also consider the total amount of caffeine from all sources of beverages and food (e.g. coffee, tea, chocolate, etc.) within an individual’s tolerance limit, to make sure excessive amounts are not consumed. Additionally, it is generally recommended that caffeine containing products should not be consumed by pregnant or lactating women, or women trying to get pregnant, as well as individuals sensitive to caffeine [17].

### Taurine

Taurine is a naturally occurring amino sulfonic acid derived from the metabolism of methionine and cysteine and is added to ED due to its role in energy metabolism, cellular osmolality, hydration, and exercise performance [19-21]. It has been reported to exhibit anti-inflammatory properties and protect against various neurotoxic insults [22], as well as influencing energy metabolism in skeletal muscle, adipose tissue, liver, and other tissues [23]. Taurine has been investigated for its effects on exercise-related outcomes [24-27]. A meta-analysis of ten studies reported that acute or chronic taurine ingestion of 1 – 6 g∙d^−1^ improves endurance exercise performance [28]. Furthermore, a recent systematic review identified support for improved VO_2_max, time-to-exhaustion, time-trial performance, anaerobic performance, muscle damage, and cognitive benefits following taurine supplementation [29]. Previous research has also indicated that taurine may influence the cardiovascular system through improved lipid profiles, modulation of calcium, antioxidant effects and antagonism of angiotensin II action [30]. Interestingly, select evidence supports the potential for taurine to reduce the potential for adverse cardiovascular symptoms associated with caffeine intake [31], which could indicate benefits of taurine’s inclusion in caffeinated ED and ES products. Additionally, taurine exerted effects on a variety of metabolites, including lactate, creatine kinase, inflammatory compounds, and markers of carbohydrate and fat oxidation. Nonetheless, inconsistencies and limitations of the present body of research on taurine have been acknowledged [29,32]. The overall prevalence of taurine in bestselling ED and ES has been reported at 37.3%, while a mean amount was not able to be calculated due the amounts not being disclosed [2]. However, existing data do not indicate safety concerns of taurine supplementation, with doses of 10 g∙d^−1^ for 6 months and 1 to 6 g∙d^−1^ for up to one year being investigated [22]. Therefore, it is unlikely that the doses of taurine in popular ED and ES products represent a safety concern. While some ED and ES products may contain a potentially ergogenic dose of ≥1 g, few products state the taurine dose explicitly.

### Ginseng

Ginseng is an extract and herbal medication derived from the roots of the plant *Panax ginseng*, although the term is also used generically for botanicals from the genus *Panax* [11,33]. It is also noteworthy that some distinct botanical compounds, such as Siberian or Russian ginseng (*Eleutherococcus senticosus*), are unrelated to *Panax ginseng* despite shared use of the term ‘ginseng’. Ginseng has long been used in traditional Chinese medicine for the improvement of stamina and vitality, while empirical research has investigated its effects on a variety of outcomes, including psychomotor performance, physical performance, circulatory function, glucose metabolism, erectile dysfunction, and immunomodulation, among others [11,34]. Most trials do not support a beneficial effect of *Panax ginseng* [34-37] or Siberian ginseng [38] on relevant exercise performance outcomes, including aerobic capacity, peak power output, and time to exhaustion. However, *Panax* ginseng and *Panax quinquefolius* (American ginseng) may alleviate fatigue in fatigued individuals at doses of 80 to 2000 mg∙d^−1^, with the optimal dose being uncertain [39]. Other research has supported acute doses of 200 to 400 mg for mood-related outcomes [40]. The overall prevalence of ginseng in bestselling ED and ES has been reported at 30.7%, while a mean content and prevalence was not able to be calculated due to the lack of dose disclosed [2]. In isolation, *Panax ginseng* exhibits few adverse events or drug interactions, although some multi-ingredient products containing ginseng have demonstrated increased adverse events [41]. Possible drug interactions between *Panax ginseng* and warfarin, phenelzine, and alcohol have also been reported [41].

### Guarana

Guarana (*Paullinia cupana*) is used as an herbal source of naturally occurring caffeine [42]. The caffeine content of guarana seeds is estimated to range from 2.5 to 6.0%, which is higher than coffee, tea, and Yerba mate [43]. As such, guarana contributes to the overall caffeine content of ED products and their stimulatory effects. Due to its caffeine content, guarana may increase fat oxidation [44], potentially augmenting fuel utilization during acute activities; however, this does not appear to translate to meaningful changes in body composition following long-term consumption. However, evidence of this effect in humans is lacking. While few investigations have administered guarana in isolation, select studies support improved cognitive performance surrounding exercise with ingestion or mouth rinsing with multi-ingredient preparations containing 112 to 300 mg of guarana per serving (when disclosed) [45-47]. The overall prevalence of guarana in bestselling ED and ES has been reported at 25.3% while a mean dosage amount was not able to be calculated due to the amounts not being disclosed [2]. Safety of guarana is largely related to its contribution to a cumulative caffeine dose, indicating safety data for caffeine itself is useful in the safety evaluation of guarana [11,15,16,48,49], although geographic and agricultural factors may influence caffeine content beyond the expected variation based on the plant tissue being considered [43].

### Carnitine

Carnitine is a nitrogenous compound that plays a critical role in the shuttling of long-chain fatty acids from the cytoplasm to the mitochondrial matrix [50]. Based on this mechanism, carnitine supplementation has been widely examined for its effect on substrate utilization, along with its putative roles in attenuating muscle damage and aiding recovery after exercise [51-53]. However, as detailed in another *International Society of Sports Nutrition* position stand [32], carnitine does not appear to exert notable effects on muscle carnitine content, fat metabolism, exercise performance, or weight loss [54-56]. The overall prevalence of carnitine in bestselling ED and ES has been reported at 16.0% [2], while a mean amount was not able to be calculated due the amounts not being disclosed. Short-term data support the safety of supplementation with up to 3 g∙d^−1^of L-carnitine L-tartrate for three weeks in healthy adults [52]. However, it does not appear that many ED or ES contain sufficient amounts of L-carnitine L-tartrate, in terms of an efficacious dose, or more commonly, do not disclose the specific amount (Tables 1-2), therefore no potential benefit from the ingredient in ED can be established.

### Choline

Choline comprises a family of water-soluble ammonium compounds, is a constituent of lecithin, and is an essential precursor for acetylcholine production [57]. It also functions as a methyl donor and is involved in lipid metabolism and cell membrane signaling [57,58]. It has been posited that exercise increases the demand for choline and that prolonged, strenuous exercise may deplete circulating choline, thus increased dietary intake of foods (e.g. eggs, meat) or dietary supplements containing choline may offer an ergogenic effect [59]. A limited number of trials have failed to support an ergogenic effect of choline supplementation, which appear to be contingent on the type and duration of exercise that would be required to result in a depletion of choline levels [60-63]. The overall prevalence of choline in bestselling ED and ES has been reported at only 2.7%, with an average content (mean ± SD) of 267 ± 330 mg∙serving^−1^ [2], which is below the dose of ~2 g commonly indicated for an ergogenic benefit, although it has been recognized that the minimum effective dose in athletes is unknown [63]. While choline can be synthesized endogenously, it is generally considered an essential nutrient due to the insufficient rate of production [58]. The Food and Nutrition Board (FNB) of the Institute of Medicine has established Adequate Intakes (AIs) and Tolerable Upper Intake Levels (ULs) for choline. The AI for adult males is 550 mg∙d^−1^, while the AI for adult females ranges from 425 to 550 mg∙d^−1^, depending on pregnancy and lactation status [58]. The UL for all adults has been set at 3,500 mg∙d^−1^ based on the potential for hypotension, liver toxicity, and other adverse effects [57].

### Vitamin B1 (Thiamin)

Thiamin is a water-soluble B vitamin involved in carbohydrate, amino acid, and lipid metabolism [64]. As thiamine pyrophosphate, this vitamin functions as an essential cofactor for many enzymatic reactions, including those which occur inside the pyruvate dehydrogenase complex such pyruvate decarboxylase and branched-chain amino acid dehydrogenase. As such, there is an interest in whether these metabolic roles warrant supplementation in active individuals. However, limited information has indicated that thiamin supplementation does not improve exercise capacity when athletes are found to have a normal dietary intake [32,65,66]. Additionally, the overall prevalence of vitamin B1 in bestselling ED and ES is only 1.3%, with an average content of 25% of the Daily Value (DV) per serving, corresponding to ~0.3 mg [2]. The FNB of the Institute of Medicine has established a Recommended Dietary Allowance (RDA) of 1.2 mg∙d^−1^ for adult males and 1.1 to 1.4 mg∙d^−1^ for adult females, depending on pregnancy and lactation status [64]. The FDA within the United States has set a DV of 1.2 mg for thiamin. Even at high doses, this vitamin does not appear to produce toxicity, perhaps due to a decline in absorption and an increase in urinary excretion. Due to a lack of adverse effect reports, even at high doses of thiamin, the FNB has not established an upper limit for thiamin [64].

### Vitamin B2 (Riboflavin)

Riboflavin is a water-soluble B vitamin that serves as a component of the coenzymes flavin mononucleotide (FMD) and flavin adenine dinucleotide (FAD), which are required for enzymatic conversions involving vitamins B3 and B6 [67]. Despite these important metabolic roles, dietary riboflavin likely exerts little to no influence on exercise capacity in the absence of prior deficiency [32,65]. The overall prevalence of vitamin B2 in bestselling ED and ES has been reported at 8.0%, with an average content (mean ± SD) of 133 ± 81 % of DV per serving, corresponding to ~1.7 mg [2]. The FNB of the Institute of Medicine has established an RDA of 1.3 mg∙d^−1^ for adult males and 1.1 to 1.6 mg∙d^−1^ for adult females, depending on pregnancy and lactation status [67]. The FDA has set a DV of 1.3 mg for riboflavin. Even at very high doses, this vitamin does not appear to produce toxicity. Due to a lack of adverse effect reports, even at high doses of riboflavin, the FNB has not established a UL for this vitamin [67].

### Vitamin B3 (Niacin)

Niacin is the generic name for a group of related compounds, including nicotinic acid, nicotinamide, and others [68]. The principal metabolically active form of this water-soluble B vitamin is the coenzyme nicotinamide adenine dinucleotide (NAD), which is required for the activity of over 400 enzymes [68]. NAD plays an essential role in catabolic reactions for all the macronutrients through its involvement in redox reactions and shuttling high-energy electrons to the electron transport chain. Based on its numerous roles and intimate involvement in energy metabolism, there is considerable interest in niacin from a sports nutrition perspective. However, limited evidence indicates a reduction in exercise capacity and blunting of fatty acid mobilization with high-dose (280 mg) niacin supplementation, potentially due to inhibition of adenylate cyclase activity and intracellular cAMP concentrations, and the subsequent decrease in hormone-sensitive lipase activity and adipocyte lipolysis [32,69]. Nonetheless, along with vitamins B6 and B12, niacin is one of the most common B vitamins in ED and ES. The overall prevalence of niacin in bestselling ED and ES has been reported at 66.7%, with an average content (mean ± SD) of 121 ± 70 % of DV per serving, corresponding to ~19.4 mg [2]. The FNB of the Institute of Medicine has established an RDA of 16 mg of niacin equivalents (NE) for adult males and 14 to 18 mg NE for adult females, depending on pregnancy and lactation status [68]. One NE is equivalent to 1 mg niacin or 60 mg of the amino acid tryptophan, which can be converted to niacin in the body. The FDA has set a DV of 16 mg for niacin. Unlike several other B vitamins, a UL has been established for niacin. The UL for all adults is 35 mg unless administered under medical supervision [68]. It has been reported that 30 to 50 mg of nicotinic acid produces skin flushing, burning, tingling, and itching due to vasodilation of subcutaneous blood vessels [68]. In contrast, nicotinamide does not produce skin flushing or other characteristic adverse effects observed with nicotinic acid. Nonetheless, the stated UL includes all sources of niacin (i.e. both nicotinic acid and nicotinamide).

### Vitamin B5 (Pantothenic Acid)

Pantothenic acid is a water-soluble B vitamin involved in several notable metabolic reactions, including the synthesis of coenzyme A [70]. Coenzyme A is essential for numerous anabolic and catabolic processes in the body. However, there is little research support for an ergogenic effect of supplementation with coenzyme A, or pantothenic acid at doses of up to 6 g∙d^−1^ [32,66,71]. Pantothenic acid is present in a moderate proportion of ED and ES, with a reported prevalence of 37.3% in bestselling products and an average content (mean ± SD) of 114 ± 77 % of DV per serving, corresponding to ~5.7 mg [2]. The FNB of the Institute of Medicine has established an AI of 5 mg for adult males and 5 to 7 mg for adult females, depending on pregnancy and lactation status [70]. The FDA has set a DV of 5 mg for pantothenic acid. This vitamin does not appear to cause toxicity even at high intakes, although extremely high intakes may cause gastrointestinal distress. Due to a lack of adverse effect reports, even at high doses of pantothenic acid, the FNB has not established a UL for this vitamin [70].

### Vitamin B6

Vitamin B6 is the generic name for a group of six water-soluble vitamer compounds: pyridoxine, pyridoxine 5′-phosphate (PNP), pyridoxal, pyridoxal 5′-phosphate (PLP), pyridoxamine, and pyridoxamine 5′-phosphate (PMP), with some sources also including a seventh member in the form of the catabolite 4-pyridoxic acid (PA) [72]. Collectively, vitamin B6 functions in over 100 enzymatic reactions, many of which concern the metabolism of proteins [73]. Vitamin B6 intake has been positively related to select physical performance metrics in healthy older adults [74,75, 76], although there is limited evidence to suggest an ergogenic effect of supplementation for exercise performance [32,65]. Nonetheless, similar to other B vitamins, the metabolic functions of vitamin B6 generate interest in the sports nutrition market. The prevalence of vitamin B6 in bestselling ED and ES has been reported at 72.0%, higher than any other B vitamin with an average content (mean ± SD) of 367 ± 648 % of DV per serving, corresponding to ~6.2 mg [2]. The FNB of the Institute of Medicine has established an RDA of 1.3 to 1.7 mg for adult males, depending on age, and 1.3 to 2.0 mg for adult females, depending on age and pregnancy/lactation status [73]. The FDA has set a DV of 1.7 mg∙d^−1^ for vitamin B6. The UL from food and supplements for all adults has been set at 100 mg, although the limit does not apply to individuals receiving higher vitamin B6 doses under medical supervision [73]. The UL was developed based on a conservative consideration of intakes that could potentially contribute to sensory neuropathy.

### Vitamin B9 (Folate)

Folate, sometimes referred to as vitamin B9, is the generic term for naturally occurring folates, including folic acid [77]. This water-soluble B vitamin is involved in nucleic acid synthesis and amino acid metabolism, among other functions. Increased dietary intake has not been found to influence exercise performance in the absence of suboptimal intake [32], although folate deficiency may impair physical performance [78]. The prevalence of folate in bestselling ED and ES has been reported at 6.7% and an average content (mean ± SD) of 258 ± 194 mcg∙serving^−1^ [2]. The FNB of the Institute of Medicine has established an RDA of 400 mcg dietary folate equivalents (DFEs) for adult males and 400 to 600 mcg DFE for adult females, depending on pregnancy and lactation status [77]. The FDA has set a DV of 400 mcg DFE for folate. The FNB did not establish a UL for folate from food due to a lack of reported adverse effects. However, a UL of 1,000 mcg has been set for folate from dietary supplements and fortified foods [77]. As folic acid, the UL of 1,000 mcg is equivalent to 1,667 mcg DFE based on the conversion between folic acid and DFE units. The UL for folate is based on concerns related to the metabolic interactions between folate and vitamin B12, particularly the potential for high folate intakes to contribute to anemia and cognitive symptoms associated with B12 deficiency [77].

### Vitamin B12

Vitamin B12 is a water-soluble B vitamin required for the development and function of the central nervous system, DNA synthesis, and red blood cell formation [79]. As the mineral cobalt is present in vitamin B12, compounds with B12 activity are referred to as cobalamins. Limited research has supported the ability of B12, as part of a three-vitamin treatment, to improve fine motor movement control and target shooting [80], an effect that could theoretically be caused by B12ʹs influence on serotonin [32]. Additionally, vitamin B12 deficiency may result in anemia and impair physical performance [78]. The prevalence of vitamin B12 in bestselling ED and ES has been reported at 66.7%, with an average content of (mean ± SD) 5,245 ± 10,475 % of DV per serving, corresponding to ~126 mcg [2]. The FNB of the Institute of Medicine has established an RDA of 2.4 mcg for adult males and 2.4 to 2.8 mcg for adult females, depending pregnancy and lactation status [79]. The FDA has set a DV of 2.4 mcg for vitamin B12. No UL has been established for B12 based on a lack of adverse effects in response to large doses.

### Vitamin C (L-ascorbic acid)

Vitamin C is a water-soluble vitamin required for the synthesis of collagen, L-carnitine, and some neurotransmitters, in addition to its role as an antioxidant and in protein metabolism [81]. Based upon these functions and its often-touted impact on the immune system, vitamin C is a popular dietary supplement among exercising and non-exercising individuals. In those who are well-nourished, supplementation with vitamin C does not appear to enhance exercise performance [32,82-84], although some data support a reduction in the incidence of upper respiratory tract infections following exercise [32,85-87]. One meta-analysis reported that prophylactic vitamin C supplementation of 250 to 1,000 mg∙d^−1^ reduced the incidence of cold by 50% in trials involving marathon runners, soldiers, and skiers exposed to intense physical exercise or cold environments [81,88]. The prevalence of vitamin C in bestselling ED and ES has been reported at 22.7%, with an average content (mean ± SD) of 59.8 ± 48.7 % of DV per serving, corresponding to ~53.8 mg [2]. The FNB of the Institute of Medicine has established an RDA of 90 mg for adult males and 75 to 120 mg for adult females, depending on pregnancy and lactation status [81]. The FDA has set a DV of 90 mg for vitamin C. For adults, a UL of 2,000 mg has been set for vitamin C from dietary supplements and foods [81]. However, the UL does not apply to individuals receiving vitamin C treatment under medical supervision.

### Vitamin D

Vitamin D is a fat-soluble vitamin that promotes calcium absorption and maintenance of blood calcium and phosphate concentrations, thereby promoting bone mineralization [89]. It also possesses other physiological roles which impact cell growth, glucose metabolism, and neuromuscular function. In foods and dietary supplements, the two main forms of vitamin D are ergocalciferol (D_2_) and cholecalciferol (D_3_), with both forms being well absorbed [90-92]. To assess vitamin D status, serum concentrations of 25-hydroxyvitamin D are commonly used, as these reflect exogenous and endogenous vitamin D. Vitamin D supplementation has not been shown to consistently enhance exercise performance [32,93], although supplementation of vitamin D and calcium may benefit bone health in some athletes [32,94]. Despite widespread interest in vitamin D supplementation in the general population, it is not a common ingredient in bestselling ED and ES, with a reported prevalence of only 2.7% [2], and an average content (mean ± SD) of 35.0 ± 21.2 % of DV per serving, corresponding to ~7 mcg; perhaps the low prevalence can be attributed to the fact that vitamin D cannot be readily absorbed without concomitant ingestion of fat, which is not a common ingredient of ED or ES. The FNB of the Institute of Medicine has established an RDA of 15 to 20 mcg (600 to 800 IU) for adult males and females [89]. The FDA has set a DV of 20 mcg (800 IU) for vitamin D. For adults, a UL of 100 mcg (4,000 IU) has been set based upon a consideration of potential adverse health effects of elevated serum vitamin D levels over time [89].

### Vitamin A

Vitamin A is a group of fat-soluble retinoid molecules (retinol, retinal, and retinyl esters) [95]. Vitamin A has several physiological functions, including its role as a component of rhodopsin in the retina, immune function, and cellular communication. Vitamin A is also formed in the body when beta-carotene is supplied in the diet. Despite these important features, vitamin A and beta-carotene supplementation have not been shown to enhance exercise performance [32,93]. The prevalence of vitamin A in bestselling ED and ES has been reported at a modest 6.7%, with an average content (mean ± SD) of 78.6 ± 86.8 % of DV per serving, corresponding to ~691 mcg [2]. The FNB of the Institute of Medicine has established an RDA of 900 mcg retinol activity equivalents (RAE) in adult males and 700 to 1,300 mcg RAE in adult females, depending on pregnancy and lactation status [95]. The FDA has set a DV of 900 mcg RAE. A UL of 3,000 mcg has also been established, although this only applies to products from animal sources and supplements containing vitamin A as retinol or retinyl esters rather than provitamin A carotenoids such as beta-carotene [95].

### Sodium

Sodium is an essential electrolyte that is present throughout the body and helps maintain extracellular volume and osmolality, along with being a vital contributor to membrane potentials and transport of numerous molecules across the cell membrane [96]. Sodium concentrations, along with other electrolytes, can change meaningfully over the course of a bout of intense exercise [97,98]. While preventing deficiencies can help maintain performance, evidence does not support performance enhancements from sodium when mineral status is adequate [32]. However, select conditions, such as initiating a training program in the heat and prolonged ultra-endurance exercise, may warrant increased sodium intake to combat hyponatremia and maintain fluid balance [32,99]. The prevalence of sodium in bestselling ED and ES has been reported at 70.7%, with an average content of (mean ± SD) 120 ± 118 mg∙serving^−1^ [2]. The FNB of the Institute of Medicine has established an AI of 1,500 mg∙d^−1^ for adults, but did not establish a UL based on insufficient evidence for sodium toxicity [96]. For product labeling, the FDA uses a DV of 2,300 mg∙d^−1^. It is recommended that sports drinks, which are often used to combat fluid and electrolyte losses during prolonged exercise, contain ~20 – 30 meq∙L^−1^ (460 – 690 mg∙L^−1^) sodium [97]. However, sports drinks and energy drinks differ in terms of both typical and recommended uses. Additionally, the sodium content of ED and ES is often due to additives containing sodium (e.g. sodium citrate, sodium benzoate, sodium bicarbonate, and salt), thereby helping to improve palatability and shelf stability, rather than the inclusion of this mineral for an ergogenic purpose.

### Potassium

Potassium is an essential electrolyte present in all body tissues, with major roles in maintaining intracellular fluid volume and membrane potential [96,100]. Physiologically, potassium and sodium are intimately linked for the functions of fluid balance, maintaining electrochemical gradients, and molecular transport. In sport, potassium has often been touted for putative anti-cramping effects, although this is uncertain due to questions regarding the etiology of cramping [32,101,102]. The prevalence of potassium in bestselling ED and ES has been reported at 34.7%, with an average content of (mean ± SD) 148 ± 197 mg∙serving^−1^ [2]. The FNB of the Institute of Medicine has established an AI of 3,400 mg∙d^−1^ for adult males and 2,600 to 2,900 mg∙d^−1^ in adult females, depending on pregnancy and lactation status [96]. However, a UL was not established based on insufficient evidence for potassium toxicity risk in the apparently healthy population [96]. For product labeling, the FDA uses a DV of 4,700 mg∙d^−1^ [100]. It is recommended that sports drinks contain ~2 – 5 meq∙L^−1^ (78 – 195 mg∙L^−1^) potassium [97]. However, as with sodium, the potassium contained in energy drinks and energy shots may not be included intentionally for ergogenic purposes but rather due to its presence in other ingredients and additives (e.g. potassium sorbate, potassium citrate monohydrate, etc.).

### Magnesium

Magnesium is a mineral that serves as a cofactor in over 300 enzyme systems [103]. These contribute to a variety of physiological processes, such as nerve and muscle function, energy production, protein synthesis, and maintenance of blood glucose. Despite the relevance of these functions for exercise, magnesium supplementation does not appear to consistently improve exercise performance in the absence of suboptimal intake [32,104,105]. However, it is noteworthy that several studies indicate lower than recommended intake of magnesium in athletes and other exercising individuals, such as military personnel [104], with select research supporting ergogenic effects of supplementation [106]. The prevalence of magnesium in bestselling ED and ES has been reported at 12.0%, with an average content of (mean ± SD) 25.4 ± 23.4 mg∙serving^−1^ [2]. The FNB of the Institute of Medicine has established an RDA of 400 to 420 mg in adult males and 310 to 360 mg in adult females, depending on pregnancy and lactation status [103]. The FDA has set a DV of 420 mg. As with other minerals, the presence of magnesium in ED and ES is typically due to its inclusion in additives or related ingredients (e.g. magnesium chloride and magnesium carbonate).

### Calcium

Calcium is the most abundant mineral in the human body and is a major structural component of bone, while also providing essential contributions for function of the neuromuscular junction, muscular contraction, and hormone secretion [107]. Supplementation with calcium may benefit populations susceptible to osteoporosis when combined with vitamin D supplementation [108], and has exhibited potential benefits for fat metabolism [109]. However, no ergogenic effects on exercise performance have been established [32]. The prevalence of calcium in bestselling ED and ES has been reported at 17.3%, with an average content of (mean ± SD) 128 ± 175 mg∙serving^−1^ [2]. The FNB of the Institute of Medicine has established an RDA of 1,000 to 1,200 mg for adults, depending on age [107]. The FDA uses a DV of 1,300 mg for labeling purposes. The calcium content of ED and ES is explained by this mineral’s presence in several additives or in conjunction with other ingredients, like vitamins (e.g. calcium chloride, calcium pantothenate, calcium disodium EDTA).

### Sugars

Sugars, including fructose, maltodextrin, etc., were traditionally present in energy drinks as a form of rapidly digestible carbohydrate. In recent years, the increased prevalence of sugar-free beverages has resulted in a prevalence of sugar of 45.3% in bestselling ED and ES, with an average content of (mean ± SD) 19.9 ± 18. g∙serving^−1^ [2]. The importance of total carbohydrate intake and timing of carbohydrate ingestion is well established for exercising populations, and the interested reader is directed to separate ISSN position stands which discuss this topic [32,110]. In brief, relevant positions adopted by the ISSN in the position statement on nutrient timing state [110]:

1. Endogenous glycogen stores are maximized by following a high-carbohydrate diet (8–12 g of carbohydrate/kg/day [g/kg/day]); moreover, these stores are depleted most by high volume exercise of moderate to high intensities.
2. If rapid restoration of glycogen is required (< 4 h of recovery time) then the following strategies should be considered:
  - aggressive carbohydrate refeeding (1.2 g/kg/h) with a preference toward carbohydrate sources that have a high (> 70) glycemic index
  - the addition of caffeine (3–8 mg∙kg BW^−1^)
  - combining carbohydrates (0.8 g∙kg hr^−1^) with protein (0.2–0.4 g∙kg hr^−1^)
3. Extended (> 60 min) bouts of high intensity (> 70% VO_2_max) exercise challenge fuel supply and fluid regulation, thus carbohydrate should be consumed at a rate of ~30–60 g of carbohydrate/h total. This can be accomplished by consuming a 6–8% carbohydrate-electrolyte solution (6–12 fluid ounces) every 10–15 min throughout the entire exercise bout, particularly in those exercise bouts that span beyond 70 min. When carbohydrate delivery is inadequate, adding protein may help increase performance, ameliorate muscle damage, promote euglycemia and facilitate glycogen re-synthesis.
4. Carbohydrate ingestion throughout resistance exercise (e.g. 3–6 sets of 8–12 repetition maximum [RM] using multiple exercises targeting all major muscle groups) has been shown to promote euglycemia and higher glycogen stores. Consuming carbohydrate solely or in combination with protein during resistance exercise increases muscle glycogen stores, ameliorates muscle damage, and facilitates greater acute and chronic exercise training adaptations.

Accordingly, ED or ES containing sugar can contribute to total and peri-exercise carbohydrate intake, although they should not be considered a primary source of carbohydrate based on the modest content of most beverages relative to needs for exercising individuals. Further, post-exercise carbohydrate co-ingested with caffeine has been shown to augment glycogen repletion [111]. Another consideration is that many ED are ~14% solutions, athletes may benefit from diluting the beverage to get within the 6-8% recommended amounts [112] which is common practice for endurance athletes using soft drinks. While there is no DV for total sugar, the FDA now utilizes a DV of 50 g/day for added sugars on Nutrition Facts labels. This labeling emphasis on added sugars reflects the *Dietary Guidelines for Americans* recommendations to limit calories from added sugars to <10% of total daily calories [113]. However, it should be noted that these recommendations are for health promotion in the general population, and athletes have specific dietary needs for performance and recovery that may differ from some stated recommendations. Moreover, consumption of high glycemic beverages may elicit hypoglycemic-like effects in healthy, non-diabetic adults [114], indicating people may want to exercise caution when considering the timing of beverage consumption. Less active individuals should consider the impact of regularly ingesting ED or ES containing high glycemic index carbohydrates on metabolic health, blood glucose, and insulin levels.

### Artificial sweeteners

Artificial sweeteners are common food and drink additives which provide sweet flavor without the calories associated with traditional sweetening agents such as sugars [115]. These compounds include non-nutritive sweeteners (NNS), which contain less than 2% of the calories of an equivalent amount of sugar, as well as nutritive sweeteners (NS), which contain more than 2% of the calories of a similar amount of sugar [116]. Also known as high-intensity sweeteners, NNS and NS are often many times sweeter than table sugar and thus are typically only added to a product in small amounts to achieve the same level of perceived sweetness as a relatively larger amount of sucrose [116]. Common artificial sweeteners approved for use or generally recognized as safe in foods and beverages by the US FDA include aspartame, acesulfame potassium, sucralose, as well as steviol glycosides (stevia), and Siraitia grosvenorii (monk fruit) extract [115]. A variety of NS including sugar alcohols like erythritol are also commonly added to products to provide sweet flavor without increased sugar content [115]. Of the 75 bestselling ED and ES products reported by Jagim and colleagues [2], 76% contained at least one artificial sweetener. Of the different sweeteners, sucralose was present in 57% of the products, followed by acesulfame potassium (33% prevalence), stevia (17% prevalence), erythritol (12% prevalence), monk fruit extract (7%), xylitol (1%), and aspartame (1%). Based on current FDA policy and expert consensus, the evidence supports the safety of these ingredients when ingested at doses common to beverages [115-118]. However, it should be noted that some people may experience gastrointestinal side effects from artificial sweetener consumption, namely from sugar alcohols like erythritol, which have been associated with digestive problems such as diarrhea (5). Otherwise, these additives appear to be safe for human consumption and have not been shown to be associated with adverse events or elevated risks of cancer, cardiovascular disease, and neurological symptoms [115-118]. In fact, the inclusion of artificial sweeteners and exclusion of sugars in relevant product formulations may help attenuate the hyper- or hypoglycemic responses resulting from the consumption of high glycemic index beverages and reduce unwanted caloric intake.

### Tyrosine

Tyrosine is a nonessential proteinogenic amino acid that serves as the precursor for several neurotransmitters, including dopamine, epinephrine, and norepinephrine [119]. A limited amount of evidence supports benefits of tyrosine supplementation for improving cognitive function during demanding cognitive or physical tasks at acute doses of 2 up to ~12 g, when disclosed [120-123]. The prevalence of tyrosine in bestselling ED and ES has been reported at 22.7% while a mean amount was not able to be calculated due the amounts not being disclosed [2].

### L-Theanine

L-theanine is a non-proteinogenic amino acid that is relatively uncommon in the diet but is found in tea and available as a dietary supplement [121]. This amino acid may aid cognitive function, with some evidence indicating a potential synergy with caffeine at doses of ~100 to 250 mg theanine and ~40 to 160 mg of caffeine [120,124-127]. Coingestion of caffeine and theanine in tea, along with their related neurochemical effects, may support this synergistic relationship, although underlying mechanisms have not been fully established [120]. The prevalence of L-theanine in bestselling energy drinks and energy shots has been reported at 17.3% while a mean amount was not able to be calculated due the amounts not being disclosed [2].

In summary, although ED and ES contain several nutrients that are purported to affect mental and/or physical performance, the primary ergogenic nutrients in most ED and ES based upon scientific evidence appear to be caffeine and/or the carbohydrate provision. Moreover, while the ergogenic value of caffeine and carbohydrates on mental and physical performance have been well-established, the potential additive benefits of other nutrients contained in ED and ES remain to be determined; largely due to the lack of disclosure regarding specific dosages of key ingredients or a failure to reach an ergogenic threshold required for that ingredient.

## 4. Acute exercise performance

A wide variety of investigations have examined the acute impact of ED and ES consumption on exercise performance, the majority of which have been published since the previous ISSN energy drink position stand was released in 2013 [1]. Based on the current body of evidence, it appears that acute consumption of these products may improve force and power production, anaerobic capacity, muscular endurance, and endurance exercise performance in athletic populations [128], though findings are quite mixed both in terms of significance and magnitude for many outcomes. The results of each study should be interpreted carefully and in context of the varied study participant populations, exercise modalities, and product ingredient profiles used in these investigations (Tables 4-9). Moreover, the mixed results of studies may be influenced by the dose, habituation of caffeine, and genetic profile of participants; all of which have been known to influence the ergogenic potential and metabolic effects of caffeine [13,129,130]. The reader should also be reminded that this position stand summarizes the available evidence surrounding ED and ES, both of which are ready-to-drink products with relatively similar marketing claims and ingredients. Because powdered energy products, such as thermogenic (‘fat-burning’) products, and multi-ingredient pre-workout supplements typically have different ingredient profiles, purported marketing claims, and usage patterns, they have largely been excluded from discussion. For more information about these products, interested readers should consult an excellent review by Jeukendrup and colleagues [131], who provides an in-depth overview of ingredients commonly found in thermogenic products, as well as a recent review by Harty et al. [132], which outlines the current body of knowledge regarding multi-ingredient pre-workout supplements.

**Table 4.** Force and Power Production Outcomes in Acute Energy Drink and Energy Shot Studies.

Study	Subjects	Design	ED/ES Condition	Timing	Performance Testing Protocol	Results	Ref #
Del Coso et al. 2012a	19 semiprofessional soccer players (21 ± 2 y, 67 ± 2 kg)	Randomized, double-blind, placebo-controlled, crossover study	ED (Sugar-free Red Bull®) providing 3 mg∙kg BW^−1^ caffeine vs. placebo	60 min prior to exercise testing	15s maximal jump test	↑ Jump height	[135]
Del Coso et al. 2012b	12 active participants (30 ± 7 y, 69 ± 10 kg,)	Randomized, double-blind, placebo-controlled, crossover study	ED (Fure®, ProEnergetics at 1 and 3 mg∙kg BW^−1^ caffeine, taurine 2000 mg, sodium bicarbonate 500 mg, L-carnitine 200 mg, and maltodextrin 705 mg) vs. the same drink without caffeine	60 min prior to exercise testing	Half-squat and BP power production with loads from 10 to 100% of 1RM	Ingestion of 1 mg/kg of caffeine did not affect maximal power during the power-load tests with respect to the placebo. 3 mg/kg ↑ maximal power in the half-squat compared to placebo condition (3 mg/kg: 2726 ± 167 vs. Placebo: 2549 ± 161 W) and bench-press (3 mg/kg: 375 ± 33 vs. Placebo: 358 ± 35 W).	[141]
Sünram-Lea et al. 2012	81 firefighter trainees (26 ± 10 y, 80.6 ± 17.1 kg)	Randomized, double-blind, placebo-controlled, crossover study	ED containing 40 mg caffeine and 50 g carbohydrates vs. ED with 80 mg caffeine and 10.25 g carbohydrates vs. placebo	75 min prior to exercise testing	Handgrip test	↑ Handgrip strength after consumption of ED containing 40 mg caffeine and 50 g carbohydrates	[148]
Eckerson et al. 2013	17 physically active men (21 ± 1 y, 85.5 ± 9.3 kg)	Randomized, double-blind, placebo-controlled, crossover study	ED (Sugar-free Red Bull®) containing 160 mg caffeine and 2000 mg taurine vs. a caffeine only drink (160 mg) vs. a non-caloric placebo	60 min prior to exercise testing	1RM BP	↔ 1RM BP	[151]
Del Coso et al. 2013a	16 elite female rugby players (23 ± 2 y, 66 ± 7 kg)	Randomized, double-blind, placebo-controlled, crossover study	ED (Fure®, ProEnergetics: 3 mg∙kg BW^−1^ caffeine, taurine 2000 mg, sodium bicarbonate 500 mg, L-carnitine 200 mg, and maltodextrin 705 mg) vs. placebo	60 min prior to exercise testing	15s maximal jump test	↑ Power output during jump test	[169]
Abien-Vicen et al. 2014	16 young basketball players (14.9 ± 0.8 y, 73.4 ± 12.4 kg)	Randomized, double-blind, placebo-controlled, crossover study	ED (Fure®, ProEnergetics: 3 mg∙kg BW^−1^ caffeine, taurine 2000 mg, sodium bicarbonate 500 mg, L-carnitine 200 mg, and maltodextrin 705 mg) vs. placebo	60 min prior to exercise testing	CMVJ test 15s maximal jump test	↑ CMVJ jump height (38.3 ± 4.4 vs. 37.5 ± 4.4 cm) ↑ Jump height in 15s jump test (30.2 ± 3.4 vs. 28.8 ± 3.4 cm).	[134]
Del Coso et al. 2014	15 male volleyball players (21.8 ± 6.9 y, 66 ± 7 kg)	Randomized, double-blind, placebo-controlled, crossover study	ED (Fure®, ProEnergetics: 3 mg∙kg BW^−1^ caffeine, taurine 2000 mg, sodium bicarbonate 500 mg, L-carnitine 200 mg, and maltodextrin 705 mg) vs. placebo	60 min prior to exercise testing	Squat Jump CMVJ test 15s maximal jump test	↑ Mean jump height for squat jumps (32.7 ± 4.2 vs. 31.1 ± 4.3 cm) ↑ CMVJ height (37.7 ± 4.4 vs. 35.9 ± 4.6 cm) ↑ Jump height in 15s jump test (30.5 ± 4.6 vs. 29.0 ± 4.0 cm)	[136]
Goel et al. 2014	15 male volleyball players (21.8 ± 6.9 y, 66 ± 7 kg)	Randomized, double-blind, placebo-controlled, crossover study	ED (Red Bull®) providing 2 mg∙kg BW^−1^ caffeine vs. placebo	60 min prior to exercise testing	Handgrip test	↔ Handgrip strength	[149]
Kammerer et al. 2014	14 male soldiers	Randomized, double-blind, placebo-controlled, crossover study	ED (Red Bull®) containing 80 mg caffeine and 1000 mg taurine vs. drink containing 80 mg caffeine vs. drink containing 1000 mg taurine vs. drink containing 80 mg caffeine and 1000 mg taurine vs. placebo	Information unavailable	Handgrip test Vertical jump test	↔ Handgrip strength ↔ Vertical jump height	[145]
Lara et al. 2014	18 female soccer players (21 ± 2 y, 57.8 ± 7.7 kg)	Randomized, double-blind, placebo-controlled, crossover study	ED (Fure®, ProEnergetics: 3 mg∙kg BW^−1^ caffeine, taurine 2000 mg, sodium bicarbonate 500 mg, L-carnitine 200 mg, and maltodextrin 705 mg) vs. placebo	60 min prior to exercise testing	CMVJ test	↑ CMVJ height (27.4 ± 3.8 vs. 26.6 ± 4.0 cm)	[138]
Abian et al. (2015)	16 male elite badminton players (25.4 ± 7.3 y, 71.8 ± 7.9 kg)	Randomized, double-blind, placebo-controlled, crossover study	ED (Fure®, ProEnergetics: 3 mg∙kg BW^−1^ caffeine, taurine 2000 mg, sodium bicarbonate 500 mg, L-carnitine 200 mg, and maltodextrin 705 mg) vs. placebo	60 min prior to exercise testing	Squat Jump CMVJ test Handgrip test	↑ Squat jump height (36.4 ± 4.3 vs. 34.5 ± 4.7 cm) and squat jump peak power ↑ CMVJ height (39.5 ± 5.1 vs. 37.7 ± 4.5 cm) and CMVJ peak power ↔ Handgrip strength	[133]
Lara et al. 2015	14 male sprint swimmers (20.2 ± 2.6 y, 73.9 ± 8.3 kg)	Randomized, double-blind, placebo-controlled, crossover study	ED (Fure®, ProEnergetics: 3 mg∙kg BW^−1^ caffeine, taurine 2000 mg, sodium bicarbonate 500 mg, L-carnitine 200 mg, and maltodextrin 705 mg) or placebo	60 min prior to exercise testing	CMVJ test Handgrip test	↑ CMVJ height (50.9 ± 5.2 vs. 49.4 ± 5.3 cm) ↑ Handgrip strength with the right hand (481 ± 49 vs. 498 ± 43 N) but not left hand	[139]
Gallo-Salazar et al. 2015	14 young elite-level tennis players (16 ± 1 y)	Randomized, double-blind, placebo-controlled, crossover study	ED (Fure®, ProEnergetics: 3 mg∙kg BW^−1^ caffeine, taurine 2000 mg, sodium bicarbonate 500 mg, L-carnitine 200 mg, and maltodextrin 705 mg) vs. placebo	60 min prior to exercise testing	Handgrip test	↑ Handgrip strength (4.2% ± 7.2%) following consumption of ED compared to placebo	[147]
Perez-Lopez et al. 2015	13 elite female volleyball players (25.2 ± 4.8 y, 64.4 ± 7.6 kg)	Randomized, double-blind, placebo-controlled, crossover study	ED (Fure®, ProEnergetics: 3 mg∙kg BW^−1^ caffeine, taurine 2000 mg, sodium bicarbonate 500 mg, L-carnitine 200 mg, and maltodextrin 705 mg) vs. placebo	60 min prior to exercise testing	Spike jump Blocking jump Squat jump CMVJ test Manual dynamometry	↑ Squat jump height (29.4 ± 3.6 vs. 28.1 ± 3.2 cm) ↑ CMVJ height (33.1 ± 4.5 vs. 32.0 ± 4.6 cm) ↑ Spike jump height (44.4 ± 5.0 vs. 43.3 ± 4.7 cm) ↑ Blocking jump height (36.1 ± 5.1 vs. 35.2 ± 5.1 cm) ↔ Manual dynamometry	[140]
Campbell et al. 2016	19 college-aged males and females (22.4 ± 3.2 y, 69.0 ± 12.7 kg)	Randomized, double-blind, placebo-controlled, crossover study	ED (VPX Redline®, Power Rush: Caffeine Anhydrous 175 mg, Vitamin C 60 mg, Niacin 5 mg, Vitamin B12 0.0625 mg, Vitamin B6 0.75 mg, Folic Acid 0.2 mg, N-Acetyl-L-Tyrosine 125 mg, Beta-Alanine 12.5 mg, DL-Phenylalanine 0.325 mg, L-Phenylalanine 0.325 mg) vs. placebo	30 min prior to exercise testing	CMVJ test	↔ CMVJ height	[142]
Astley et al. 2018	15 resistance-trained males (21.0 ± 0.3 y; 79.6 ± 1.8 kg)	Randomized, double-blind, placebo-controlled, crossover study	ED (caffeine (64 mg/200 mL) [2.5 mg∙kg BW^−1]^ caffeine, soda water, carbohydrates 71 g, taurine (800 mg/200 mL), glucoronolactone (48 mg/200 mL), inositol (40 mg / 200 mL), natural extract of guarana and vitamins (B3, B5, B2, B6, B12) vs. placebo	60 min prior to exercise testing	BP RTF (80% 1RM) Unilateral knee extension RTF (80% 1RM) Handgrip test	↑ BP repetitions (10.2 ± 0.4 reps vs. 8.1 ± 0.5 reps) ↑ Unilateral knee extension repetitions (11.5 ± 0.9 vs. 9.5 ± 0.8 reps) ↑ handgrip strength in the right (53.7 ± 1.5 vs. 47.7 ± 1.6 kg) and left hand (52.9 ± 1.5 vs. 45.9 ± 1.3 kg)	[150]
Jacobson et al. 2018	36 male and female undergraduate students (23.07 ± 2.36 y, 76.9 ± 16.7 kg)	Randomized, double-blind, placebo-controlled, crossover study	ES (5-hour ENERGY, Living Essentials: 57 ml containing 240 mg caffeine, taurine, glucuronolactone, malic acid, N-Acetyl L tyrosine, L-phenylalanine, and citicoline labeled as “Energy Blend – 2000 mg”.) vs. placebo	30 min prior to exercise testing	CMVJ test	↔ CMVJ height	[144]
Chtorou et al. 2019	19 male physical education students (21.2 ± 1.2 y, 76.6 ± 12.6 kg)	Randomized, double-blind, placebo-controlled, crossover study	ED (Red Bull®: 160 mg caffeine and 2 g taurine, 1.2 g glucuronolactone, 54 g carbohydrate, 40 mg niacin, 10 mg pantothenic acid, 10 mg vitamin B6, and 10 μg vitamin B12) vs. placebo	60 min prior to exercise testing	Handgrip test	↑ Handgrip strength (58.2 ± 2.4 vs. 55.5 ± 2.7 kg)	[146]
Harty et al. 2020	16 resistance-trained males (n = 8; 22.4 ± 4.9 y, 78.8 ± 14.0 kg) and females (n = 8; 24.5 ± 4.8 y, 67.5 ± 11.9 kg)	Randomized, double-blind, placebo-controlled, crossover study	ED (Bang® Keto Coffee: 130 kcal, 300 mg caffeine, 20 g protein) vs. placebo (30 kcal, 11 mg caffeine, 1 g protein)	40 min prior to exercise testing	LP 1RM Maximal isometric and isokinetic squat testing	↔ LP 1RM ↔ Maximal isometric/isokinetic squat performance	[152]

↑ = ED/ES significantly greater (p < 0.05) than control; ↓ = ED/ES significantly less (p < 0.05) than control; ↔ = no significant difference between ED/ES and control; 1RM = 1 repetition maximum; BP = Bench press; CMVJ = Countermovement vertical jump; ED = energy drink; ES = energy shot; g = gram; kg = kilogram; LP = Leg press; mg = milligram; RTF = repetitions to fatigue; y = years; μg** **= microgram

**Table 5.** Muscular Endurance and Anaerobic Capacity Outcomes in Acute Energy Drink and Energy Shot Studies.

Study	Subjects	Design	ED/ES Condition	Timing	Performance Testing Protocol	Results	Ref #
Alford et al. 2001	12 healthy male (n = 7) and female (n = 5) subjects, aged 18–30 years; mean of ~23 years	Randomized, double-blind, placebo-controlled, crossover study	ED (Red Bull®) containing 80 mg caffeine, 1 g taurine, 600 mg glucuronolactone, 27 g carbohydrates, and 40 mg inositol vs. placebo	30 min prior to exercise testing	30-s WAnT	↑ Time maintained at maximal speed (7.1 ± 0.3 s vs. 5.7 ± 0.4 s)	[161]
Forbes et al. 2007	15 healthy males (n = 11) and females (n = 4) (21 ± 5 y)	Randomized, double-blind, placebo-controlled, crossover study	ED (Red Bull®) providing 2 mg∙kg BW^−1^ caffeine vs. isoenergetic placebo	60 min prior to exercise testing	3 sets BP RTF; 70% 1RM; 1-min rest intervals Three 30-s WAnT; 2-min rest intervals	↑ Total BP repetitions (34 ± 9 vs. 32 ± 8 reps) ↔ WAnT peak or average power	[153]
Hoffman et al. 2009	12 male strength/power athletes (21.1 ± 1.3 y; 88.6 ± 12.1 kg)	Randomized, double-blind, placebo-controlled, crossover study	ED (VPX Redline®; 158 mg of caffeine) vs. placebo	10 min prior to exercise testing	30-s WAnT	↔ WAnT performance	[158]
Campbell et al. 2010	15 recreationally active males (n = 9) and females (n = 6) (21.7 ± 1.6 y; 75.1 ± 20.2 kg)	Randomized, double-blind, placebo-controlled, crossover study	ED containing 160 mg of caffeine (~2.1 mg∙kg BW^−1^) vs. placebo	60 min prior to exercise testing	Two 20-s WAnT	↔ WAnT performance	[157]
Dawes et al. 2011	41 healthy males (21.7 ± 1.74 y; 81.2 ± 10.9 kg)	Randomized, double-blind, placebo-controlled, crossover study	ED (VPX Redline®, Power Rush: Caffeine Anhydrous 175 mg, Vitamin C 60 mg, Niacin 5 mg, Vitamin B12 0.0625 mg, Vitamin B6 0.75 mg, Folic Acid 0.2 mg, N-Acetyl-L-Tyrosine 125 mg, Beta-Alanine 12.5 mg, DL-Phenylalanine 0.325 mg, L-Phenylalanine 0.325 mg) vs. placebo	30 min prior to exercise testing	Pushup RTF	↑ Total pushup repetitions (12.5% vs. 3.25%)	[155]
Astorino et al. 2012	15 collegiate soccer players (19.5 ± 1.1 y; 63.4 ± 6.1 kg)	Randomized, double-blind, placebo-controlled, crossover study	ED (Red Bull®) providing 1.3 mg∙kg BW^−1^ caffeine and 1000 mg taurine vs. placebo	60 min prior to exercise testing	8 bouts of the modified t-test	↔ Mean sprint time ↔ HR and RPE	[160]
Duncan et al. 2012	13 resistance-trained males (22.7 ± 6.0 y)	Randomized, double-blind, placebo-controlled, crossover study	ED (179 mg caffeine alongside a matrix of the following ingredients: vitamins B3, B6, B9, and B12; tyrosine; taurine; malic acid; and glucuronolactone in a total volume of 1,024 mg combined) vs. placebo.	60 min prior to exercise testing	1 set RTF on BP, deadlift, prone row, and back squat; 60% 1RM	↑ Mean repetitions performed to failure (ED: 20.1 ± 6.3 vs. Placebo: 18.6 ± 5.6 reps)	[154]
Eckerson et al. 2013	17 physically active men (21 ± 1 y, 85.5 ± 9.3 kg)	Randomized, double-blind, placebo-controlled, crossover study	ED (Sugar-free Red Bull®) containing 160 mg caffeine and 2000 mg taurine vs. a caffeine only drink (160 mg) vs. placebo	60 min prior to exercise testing	1 set BP RTF; 70% 1RM	↔ Volume load	[151]
Campbell et al. 2016	19 college-aged males and females (22.4 ± 3.2 years, 69.0 ± 12.7 kg body mass)	Randomized, double-blind, placebo-controlled, crossover study	ED (VPX Redline®, Power Rush: Caffeine Anhydrous 175 mg, Vitamin C 60 mg, Niacin 5 mg, Vitamin B12 0.0625 mg, Vitamin B6 0.75 mg, Folic Acid 0.2 mg, N-Acetyl-L-Tyrosine 125 mg, Beta-Alanine 12.5 mg, DL-Phenylalanine 0.325 mg, L-Phenylalanine 0.325 mg) vs. placebo.	30 min prior to exercise testing	YMCA bench press test Curl up test Repeated sprint test	↔ Bench press repetitions ↔ Curl up performance ↔ Sprint speed	[142]
Magrini et al. 2016	31 healthy (males, n = 23; females, n = 8) Placebo (Age: 21.2 ± 1.7 yrs.; Height: 169.0 ± 13.2 cm; Weight: 78.6 ± 17.9 kg; Body fat%: 16.65 ± 5.6 %) and ED (Age: 23.2 ± 2.6 yrs.; Height: 167.7 ± 10.4 cm; Weight: 75.7 ± 14.9 kg; Body fat%: 14.91 ± 4.7 %)	Randomized, double-blind, placebo controlled, parallel design	ED containing 158 mg caffeine and proprietary blend of other ingredients vs. placebo	30 min prior to exercise testing	Pushup RTF	↔ Pushup repetitions	[156]
Zileli et al. 2019	18 male amateur soccer players (21.0 ± 1.6 y, 70.6 ± 9.1 kg)	Randomized, double-blind, placebo-controlled, crossover study	ED (Red Bull®) containing 80 mg caffeine, 1000 mg taurine, 600 mg glucuronolactone, 27 g carbohydrates, and 40 mg inositol vs. apple juice placebo	60 min prior to exercise testing	WAnT	↔ WAnT performance	[159]
Harty et al. 2020	16 resistance-trained males (n = 8; 22.4 ± 4.9 y; 78.8 ± 14.0 kg and females (n = 8; 24.5 ± 4.8 y; 67.5 ± 11.9 kg)	Randomized, double-blind, placebo-controlled, crossover study	ED (Bang® Keto Coffee) containing 130 kcal, 300 mg caffeine, 20 g protein vs. placebo (30 kcal, 11 mg caffeine, 1 g protein)	40 min prior to exercise testing	LP RTF; 2x body mass for males/1.5x body mass for females	↔ LP repetitions	[152]
Reis et al. 2021	12 males (22 ± 2.6 y, 74.4 ± 5.5 kg)	Randomized, double-blind, placebo-controlled, crossover study	ED providing 3 mg∙kg BW^−1^ caffeine and carbohydrates vs. sugar-free ED containing 3 mg∙kg BW^−1^ caffeine vs. placebo	40 min prior to exercise testing	RPE during 55-minute treadmill run at 65-75% VO_2_max	↑ Sprint time of 19.8% and 19.0% compared to placebo following ingestion of ED and sugar-free ED, respectively ↓ RPE during exercise for both ED conditions compared to placebo	[185]

↑ = ED/ES significantly greater (p < 0.05) than control; ↓ = ED/ES significantly less (p < 0.05) than control; ↔ = no significant difference between ED/ES and control; 1RM = 1 repetition maximum; BP = Bench press; ED = energy drink; g = gram; HR = heart rate; kg = kilogram; LP = Leg press; mg = milligram; RPE = Rating of perceived exertion; RTF = repetitions to fatigue; y = years; WAnT = Wingate anaerobic cycle test

**Table 6.** Aerobic Exercise Performance Outcomes in Acute Energy Drink and Energy Shot Studies.

Study	Subjects	Design	ED/ES Condition	Timing	Performance Testing Protocol	Results	Ref #
Geiβ et al. 1994	10 endurance athletes (24.5 ± 3.5 y, 78.8 ± 8.3 kg)	Randomized, double-blind, placebo-controlled, crossover study	ED (Red Bull®) containing taurine, glucuronolactone and caffeine (U1) vs. ED without taurine or glucuronolactone (U2) vs. placebo (U3)	After 30 min submaximal cycling, 30 minutes prior to a TTE test	TTE on a cycle ergometer at 70% VO_2_max (following 60 min cycling at ~70% VO_2_max	↑ TTE in U1 compared to U3 treatment (24.4%) ↑ TTE in U2 compared to U3 treatment (14.9%)	[163]
Alford et al. 2001	14 healthy male (n = 7) and female (n = 7) subjects (~23 y)	Randomized, double-blind, placebo-controlled, crossover study	ED (Red Bull®: 80 mg caffeine, 1000 mg taurine, 600 mg glucuronolactone, 27 g carbohydrates, and 40 mg inositol) vs. placebo	30 min prior to exercise testing	Cycling at 65-75% maximum heart rate until heart rate exceeded 75% max heart rate	↑ Cycle time	[161]
Umaña-Alavarado et al. 2005	11 male runners and triathletes (30.18 ± 11.5 y; 68.3 ± 8.1 kg)	Randomized, double-blind, placebo-controlled, crossover study	ED providing 32 mg caffeine/100 ml (6 ml∙kg BW^−1^ provided) vs. placebo	30 min prior to exercise testing	10-km run TT	↔ TT performance	[206]
Candow et al. 2009	17 physically active males (n = 9) and females (n = 8) (21 ± 4 y, 73.4 ± 3.1 kg)	Randomized, double-blind, placebo-controlled, crossover study	ED (Red Bull®) providing 2 mg/kg caffeine vs. drink with ∼147 mg caffeine only vs. placebo	60 min prior to exercise testing	Running TTE at 80% VO_2_max	↔ TTE performance ↔ Blood lactate response	[173]
Ivy et al. 2009	12 male (n = 6) and female (n = 6) trained cyclists (27.3 ± 1.7 y, 68.9 ± 3.2 kg)	Randomized, double-blind, placebo-controlled, crossover study	ED (Red Bull®: 160 mg caffeine, 2.0 g taurine, 1.2 g glucuronolactone, 54 g carbohydrate, 40 mg niacin, 10 mg pantothenic acid, 10 mg vitamin B6, and 10 μg vitamin B12 vs. placebo	40 min prior to exercise testing	TT to complete a standardized amount of work equal to 1 hr of cycling at 70% Wmax	↑ TT performance (3,690 ± 64 vs. 3,874 ± 93s). ↔ Substrate utilization	[166]
Kazemi et al. 2009	12 female student athletes (22 ± 0.6 y, 56.91 ± 6.79 kg)	Randomized, double-blind, placebo-controlled, crossover study	6 mg∙kg BW^−1^ of Phantom ED (85 mg of caffeine; 1,000 mg of taurine and 26.8 g of carbohydrates, vs. Dragon ED (50 mg of caffeine, and 28.3 g of carbohydrates) vs. placebo.	40 min prior to exercise testing	TTE on treadmill to determine VO_2_max	↑ TTE performance (both ED)	[164]
Rahnama et al. 2010	10 male student athletes (22.4 ± 2.1 y, 74.2 ± 8.5 kg)	Randomized, controlled trial with 3 conditions: Red Bull vs. Hype vs Control	ED 1 (Red Bull®) containing 85 mg caffeine, 122.5 kcals, 28.3 g carbs, 1000 mg of taurine, 600 mg glucuronolactone, & B vitamins vs. ED 2 (Hype®) containing 75 mg of caffeine, 99.1 kcals, 24.8 g carbohydrate, 1000 mg Taurine, 600 mg glucuronolactone, & B vitamins vs. placebo	40 min prior to exercise testing	TTE to determine VO_2_max	↑ VO_2_max Test (both ED; ED 1 = 52.9 ± 5.3 ml∙kg∙min^−1^, ED 2 = 51.9 ± 3.9 ml∙kg∙min^−1^, vs. placebo = 46.8 ± 4.1 ml∙kg∙min^−1^) ↑ TTE (both ED; ED 1 = 14.6 ± 1.4 min, ED 2 = 14.4 ± 1.1 min, vs. placebo = 13.0 ± 1.0 min)	[165]
Del Coso et al. 2012a	19 semiprofessional soccer players (21 ± 2 y, 67 ± 2 kg)	Randomized, double-blind, placebo-controlled, crossover study	ED (Sugar-free Red Bull®) providing 3 mg∙kg BW^−1^ caffeine vs. placebo	60 min prior to exercise testing	Running distance and speed during simulated soccer game	↑ Total distance covered at a speed greater than 13 km∙hr^−1^	[135]
Del Coso et al. 2013a	16 elite female rugby players (23 ± 2 y, 66 ± 7 kg)	Randomized, double-blind, placebo-controlled, crossover study	ED (Fure®, ProEnergetics: 3 mg∙kg BW^−1^ caffeine, taurine 2000 mg, sodium bicarbonate 500 mg, L-carnitine 200 mg, and maltodextrin 705 mg) vs. placebo	60 min prior to exercise testing	Running speed and sprint velocity during rugby sevens game	↑ Running pace at a speed greater than 13 km∙hr^−1^	[137]
Del Coso et al. 2013b	26 elite male rugby players (25 ± 2 y, 93 ± 15 kg)	Randomized, double-blind, placebo-controlled, crossover study	(Fure®, ProEnergetics: 3 mg∙kg BW^−1^ caffeine, taurine 2000 mg, sodium bicarbonate 500 mg, L-carnitine 200 mg, and maltodextrin 705 mg) vs. placebo	60 min prior to exercise testing	Running distance and speed during simulated rugby game	↑ Total distance covered ↑ Total distance covered at a speed greater than 20 km∙hr^−1^	[169]
Schubert et al. 2013	6 male runners (22.5 ± 1.8 y; 65.4 ± 10.0 kg)	Randomized, double-blind, placebo-controlled, crossover study	ES (Guayakí Yerba Maté Organic Energy Shot™) containing 140 mg caffeine vs. ES (Red Bull Energy Shot™) containing 80 mg caffeine vs. placebo	50 min prior to exercise testing	5-km TT on treadmill	↔ TT performance	[176]
Kammerer et al. 2014	14 male soldiers	Randomized, double-blind, placebo-controlled, crossover study	ED (Red Bull®: 80 mg caffeine and 1000 mg taurine) vs. drink with 80 mg caffeine vs. drink with 80 mg caffeine and 1000 mg taurine vs. drink with 1000 mg taurine vs. placebo	Information unavailable	Incremental treadmill TTE	↔ TTE performance	[145]
Lara et al. 2014	18 female soccer players (21 ± 2 y, 57.8 ± 7.7 kg)	Randomized, double-blind, placebo-controlled, crossover study	ED (Fure®, ProEnergetics: 3 mg∙kg BW^−1^ caffeine, taurine 2000 mg, sodium bicarbonate 500 mg, L-carnitine 200 mg, and maltodextrin 705 mg) vs. placebo	60 min prior to exercise testing	Simulated soccer match (2 × 40 min)	↑ Total running distance (6,631 ± 1,618 vs. 7,087 ± 1,501 m) ↑ Running distance covered at >18 km/h (161 ± 99 vs. 216 ± 103 m)	[138]
Nelson et al. 2014	14 recreationally active males and females (25.5 ± 4.1 y, 77.9 ± 18.4 kg)	Randomized, double-blind, placebo-controlled, crossover study	ED (Monster®: 2 mg∙kg BW^−1^ caffeine, 0.65 mg∙kg BW^−1^ of carbohydrates, 25 mg∙kg BW^−1^ of taurine, 5 mg∙kg BW^−1^ of pana-ginseng, 1.5 mg∙kg BW^−1^ of Vitamin C, 0.04 mg∙kg BW^−1^ of riboflavin, 0.5 mg∙kg BW^−1^ of niacin, 0.05 mg∙kg BW^−1^ of Vitamin B6 and 0.15 mg∙kg BW^−1^ of Vitamin B12 vs. placebo	60 min prior to exercise testing	Cycling TTE at 100% ventilatory threshold	↔ TTE performance	[174]
Phillips et al. 2014	11 trained male cyclists (33.4 ± 8.9 y; 81 ± 7.6 kg)	Randomized, double-blind, placebo-controlled, crossover study	ED containing 160 mg caffeine, 2 g taurine, 1.2 g glucuronolactone, 56 g carbohydrate, and B vitamins vs. cola matched for caffeine and carbohydrate vs. flavored sparkling water placebo	50 min prior to exercise testing	25-mile simulated road TT	↔ TT performance	[175]
Al-Fares et al. 2015	32 healthy college-aged females (19.9 ± 0.8 y; 51.7 ± 3.7 kg)	Single-blind, placebo-controlled, crossover design	4 mL/kg ED (containing 2.0 g taurine, 1.2 g glucuronolactone, 160 mg caffeine, 54 g carbohydrate, 40 mg niacin, 10 mg pantothenic acid, 10 mg vitamin B6, and 10 μg vitamin B12) or PL	45 min prior to exercise testing	Bruce treadmill exercise protocol	↔ TTE performance (PL, 11.7 ± 1.5 min; ED, 11.4 ± 1.6 min).	[171]
Quinlivan et al. 2015	11 trained male cyclists (31.7 ± 5.9 y, 82.3 ± 6.1 kg)	Randomized, double-blind, placebo-controlled, crossover study	ED (Red Bull®: 3 mg/kg caffeine vs. caffeine anhydrous capsule providing 3 mg∙kg BW^−1^ vs. placebo	90 min prior to exercise testing	TT equivalent to 1 h cycling at 75% peak power output	↑ TT performance compared to placebo (ED = 2.8%; capsule = 3.1%)	[168]
Gallo-Salazar et al. 2015	14 young elite-level tennis players (16.36 ± 1.15 y; 65.2 ± 1.6 kg)	Randomized, double-blind, placebo-controlled, crossover study	ED (Fure®, ProEnergetics: 3 mg∙kg BW^−1^ caffeine, taurine 2000 mg, sodium bicarbonate 500 mg, L-carnitine 200 mg, and maltodextrin 705 mg) vs. placebo	60 min prior to exercise testing	Simulated tennis singles match	↑ Running pace at high intensity (46.7 ± 28.5 vs. 63.3 ± 27.7 m∙hr^−1^)	[147]
Del Coso et al. 2016	13 elite male field hockey players (23.2 ± 3.9 y; 76.1 ± 6.1 kg)	Randomized, double-blind, placebo-controlled, crossover study	ED (Fure®, ProEnergetics: 3 mg∙kg BW^−1^ caffeine, taurine 2000 mg, sodium bicarbonate 500 mg, L-carnitine 200 mg, and maltodextrin 705 mg) vs. placebo	60 min prior to exercise testing	Simulated field hockey match (2 × 25 min)	↑ Distance covered during high intensity running ↔ Total distance covered during the game	[170]
Prins et al. 2016	18 recreational endurance runners (13 men and 5 women) (20.4 ± 3.3 y; 71.3 ± 17.2 kg)	Randomized, double-blind, placebo-controlled, crossover study	500 ml of ED (Red Bull®: 2.0 g taurine, 1.2 g glucuronolactone, 160 mg caffeine, 54 g carbohydrates (sucrose and glucose), 40 mg niacin, 10 mg pantothenic acid, 10 mg vitamin B6, and 10 mg vitamin B12) vs. placebo	60 min prior to exercise testing	5-km TT on a treadmill	↑ TT performance (Red Bull: 1,413.2 ± 169.7 vs. PLA: 1,443.6 ± 179.2 s) ↔ Distance covered at 5-minute splits	[167]
Zileli et al. 2019	18 male amateur soccer players (21.0 ± 1.6 y, 70.6 ± 9.1 kg)	Randomized, double-blind, placebo-controlled, crossover study	ED (Red Bull®: containing 80 mg caffeine, 1 g taurine, 600 mg glucuronolactone, 27 g carbohydrates, and 40 mg inositol) vs. apple juice placebo	60 min prior to exercise testing	Yo-Yo intermittent recovery test	↔ Yo-Yo test performance	[159]
Alansare et al. 2021	11 NCAA Division I middle distance runners (20.8 ± 1.5 y; 60.5 ± 10.7 kg)	Randomized, double-blind, placebo-controlled, crossover study	240 mL ED (8% calamansi juice, 10% glucose, 0.8% taurine, and 0.4% BCAA) vs. placebo	60 min prior to exercise testing	3-km running TT	↔ TT performance	[172]

↑ = ED/ES significantly greater (p < 0.05) than control; ↓ = ED/ES significantly less (p < 0.05) than control; ↔ = no significant difference between ED/ES and control; ED = energy drink; ES = energy shot; g = gram; kg = kilogram; km = kilometer; km∙hr^−1^ = kilometer per hour; m∙hr^−1^ = miles per hour; mg = milligram; y = years; BF% = Body fat percentage; TT = Time trial; TTE = time to exhaustion

**Table 7.** Metabolic Effects/Fuel Utilization in Acute Energy Drink and Energy Shot Studies.

Study	Subjects	Design	ED/ES Condition	Timing	Performance Testing Protocol	Results	Ref #
Mendel et al. 2007	10 healthy males (29.8 ± 9.2 y, 83.0 ± 10.3 kg) and females (30.6 ± 7.0 y, 69.0 ± 11.1 kg)	Randomized, double-blind, placebo-controlled, crossover study	ED (Celsius^TM^: 5 kcals, Vitamin C 60 mg, Riboflavin 1.7 mg, Niacin 20 mg, Vitamin B 2 mg, Vitamin B12, Biotin 300 mcg, Pantothenic Acid 10 mg, Calcium 50 mg, Chromium 50 mcg, Sodium 6 mg, and unspecified amounts [1.8 g total] of caffeine, taurine, guarana extract, green tea extract, glucuronolactone, and ginger extract) vs. Diet Coke®	30 min prior to testing	RMR test	↑ RMR (+13.8% at 1 h post, +14.4% greater at 2 h post, and +8.5% greater at 3 h post-ingestion)	[218]
Rashti et al. 2009	10 physically active women (20.4 ± 0.7 y, 67.0 ± 7.0 kg)	Randomized, double-blind, placebo-controlled, crossover study	ED (Meltdown RTD®: 230 mg caffeine and undisclosed amounts of botanical compounds; methyl tetradecylthioacetic acid, yerba mate extract, methyl-synephrine, methylphenylethylene, 11-hydroxy yohimbine, yohimbine HCL, alpha-yohimbine, and methyl-hordenine HCL) vs. placebo	Half ED provided at baseline and other half after 30 minutes of testing	RMR test	↑ RMR area under the curve across three hours of testing (+10.8%) ↑ RMR during hours 2 and 3 of testing	[220]
Del Coso et al. 2012b	12 active participants (30 ± 7 y, 69 ± 10 kg)	Randomized, double-blind, placebo-controlled, crossover study	ED (Fure®, ProEnergetics: 3 mg∙kg BW^−1^ caffeine, taurine 2000 mg, sodium bicarbonate 500 mg, L-carnitine 200 mg, and maltodextrin 705 mg) vs. same ED without caffeine	60 min prior to testing	RMR test	↔ Resting metabolic rate	[141]
Kinsinger et al. 2016	23 male recreational athletes (21.7 ± 3.3 y; 82.5 ± 15 kg)	Randomized, double-blind, placebo-controlled, crossover study	ES (5-Hour Energy®: 18 mg Sodium and 1870 mg of Energy Blend (Taurine, Glucuronic acid (glucuronolactone), Malic Acid, N-Acetyl L-Tyrosine, L-Phenylalanine and Citicoline) 30 mg Niacin (Niacinamide), 40 mg Vitamin B6 (Pyridoxine Hydrochloride), 400 mcg Folic Acid and 500 mcg Vitamin B12 [2000% of the DV for vitamins B6 and 8333% of the DV vitamin B12]). vs. placebo	30 min prior to exercise testing	Bruce treadmill protocol	↔ VO_2_peak ↔ Substrate utilization	[184]
Peveler et al. 2017	15 recreational male (n = 12, 21.8 ± 2.2 y, 84.0 ± 15.4 kg) and female (n = 3, 24 ± 4.4 y, 72.6 ± 6.6 kg) runners	Randomized, double-blind, placebo-controlled, crossover study	ED (Red Bull®: 248.42 mL, 80 mg of caffeine, 1,000 mg of taurine) vs. ED (Monster®: 473.18 mL, 163 mg of caffeine, 1,000 mg of taurine,) vs. ES (5-Hour Energy®: 27.5 mL, 207 mg of caffeine, 479.9 mg of taurine) vs. placebo	60 min prior to exercise testing	15 minutes running at 70% VO_2_max	↔ Running economy, VO_2_ measures or heart rate ↓RPE Post ES ingestion compared to ED.	[183]
Harty et al. 2020	16 resistance-trained males (n = 8, 22.4 ± 4.9 y, 78.8 ± 14.0 kg) and females (n = 8, 24.5 ± 4.8 y, 67.5 ± 11.9 kg)	Randomized, double-blind, placebo-controlled, crossover study	ED (Bang® Keto Coffee: 130 kcal, 300 mg caffeine, and 20 g protein vs. placebo (30 kcal, 11 mg caffeine, 1 g protein)	15 min prior to testing	RMR test	↑ RMR (+0.18 kcal∙min^−1^)	[152]
Dalbo et al. 2008	60 healthy males (n = 30, 23.2 ± 4.0 y, 81.7 ± 11.3 kg) and females (n = 30, 23.4 ± 3.1 y, 62.1 ± 9.9 kg)	Randomized, single-blind, placebo-controlled, parallel groups	ED (Celsius^TM^) containing 200 mg caffeine and unspecified amounts of taurine, guarana extract, green tea extract, glucuronolactone, and ginger extract vs. non-caloric, non-caffeinated commercially available diet soda	Consumed assigned drink after baseline measures. Assessments were repeated at 30, 60, 120, and 180 minutes after ingestion	RMR test, RER, glycerol, plasma FFA	↑ RMR at 60, 120, and 180 min after ingestion ↔ RER ↑ FFA and glycerol at 30, 60, 120, and 180 min after ingestion	[178]
Reis et al. 2021	12 males (22 ± 2.6 y, 74.4 ± 5.5 kg)	Randomized, double-blind, placebo-controlled, crossover study	ED providing 3 mg∙kg BW^−1^ caffeine and carbohydrates vs. sugar-free ED providing 3 mg∙kg BW^−1^ caffeine vs. placebo	40 min prior to exercise testing	55-minute treadmill run at 65-75% VO_2_max	↓ RER during minutes 0-5 and 40-45 of exercise in sugar-free ED condition only	[185]

↑ = ED/ES significantly greater (p < 0.05) than control; ↓ = ED/ES significantly less (p < 0.05) than control; ↔ = no significant difference between ED/ES and control; ED = energy drink; ES = energy shot; FFA = free fatty acids; g = gram; DV = daily value; h = hours; kg = kilogram; mg = milligram; min = minutes; RER = respiratory exchange ratio; RMR = resting metabolic rate; y = years; BMI = Body mass index; RPE = ratings of perceived exertion

**Table 8.** Cognitive Effects, Reaction Time, and Subjective Effects in Acute Energy Drink and Energy Shot Studies.

Study	Subjects	Design	ED/ES Condition	Timing	Performance Testing Protocol	Results	Ref #
Alford et al. 2001	14 healthy males (n = 7) and females (n = 7) (~23 y)	Randomized, double-blind, placebo-controlled, crossover study	ED (Red Bull®) containing 80 mg caffeine, 1 g taurine, 600 mg glucuronolactone, 27 g carbohydrates, and 40 mg inositol vs. placebo.	30 min prior to testing	Choice reaction time testing Subjective measures of alertness	↑ Reaction time performance ↑ Alertness	[161]
Scholey et al. 2004	20 college aged males and females (21.1 y)	Randomized, double-blind, placebo-controlled, crossover study	ED containing 75 mg caffeine, 37.5 g glucose, and flavoring herbs vs. water plus 75 mg caffeine vs. water plus 37.5 g glucose vs. water with flavoring herbs vs. placebo	30 min prior to testing	Mood assessment Cognitive testing	↑ Secondary memory performance ↑ Speed of attention ↔ Mood ↔ Reaction time performance	[187]
Umaña-Alavarado et al. 2005	11 male runners and triathletes (30.18 ± 11.5 y; 68.3 ± 8.1 kg)	Randomized, double-blind, placebo-controlled, crossover study	ED (6 mL∙kg BW^−1^ containing 32 mg caffeine/100 mL) or placebo	30 min prior to testing	RPE during 10-km run	↓ RPE during exercise	[206]
Candow et al. 2009	17 physically active university students (21 ± 4 y, 73.4 ± 3.1 kg)	Double-blind, crossover, repeated-measures study	ED (Red Bull®: 2 mg∙kg BW^−1^ caffeine, 25 mg∙kg BW^−1^ taurine, 15 mg∙kg BW^−1^ glucuronolactone, 0.45 mg∙kg BW^−1^ Niacin, 0.15 mg∙kg BW^−1^ Pantothenic acid, 0.05 mg∙kg BW^−1^ Vitamin B6, 0.04 mg∙kg BW^−1^ Riboflavin, 0.025 μg∙kg BW^−1^ Vitamin B12) vs. placebo	60 min prior to testing	RPE during run TTE test at 80% VO_2_max	↔ RPE	[173]
Hoffman et al. 2009	12 male strength/power athletes (21.1 ± 1.3 y; 88.6 ± 12.1 kg)	Randomized, double-blind, placebo-controlled, crossover study	ED (VPX Redline®; 158 mg of caffeine) vs. placebo	10 min prior to testing	Reaction time testing (Makoto device) Self-reported mood	↑ Successful contacts during reaction time trials ↑ Total percentage of successful contacts during testing. ↑ Feelings of focus and energy	[158]
Howard et al. 2010	80 adult participants (20.1 ± 3.1 y; 74-80 kg)	Randomized, double-blind, placebo-controlled, crossover study	ED (Red Bull®; 1.8 mL∙kg BW^−1^) vs. ED (Red Bull®; 3.6 mL∙kg BW^−1^) vs. ED (Red Bull®; 5.4 mL∙kg BW^−1^) vs. placebo vs. no drink	30 min prior to testing	Behavioral control test (cued no-go) Self-reported stimulation, sedation, and fatigue	↑ Reaction time (all ED conditions compared to placebo) ↑ Reaction time (in 1.8 mL∙kg BW^−1^ ED condition compared to no drink) ↑ Subjective stimulation post ED consumption (in 1.8 mL∙kg BW^−1^and 5.4 mL∙kg BW^−1^ ED conditions compared to placebo) ↓ Fatigue change scores (in all ED conditions compared to both the no drink and placebo)	[198]
Duncan et al. 2012	13 resistance-trained males (22.7 ± 6.0 y)	Randomized, double-blind, placebo-controlled, crossover study	ED (179 mg caffeine alongside a matrix of the following ingredients: vitamins B3, B6, B9, and B12; tyrosine; taurine; malic acid; and glucuronolactone in a total volume of 1,024 mg combined) vs. placebo	60 min prior to testing	RPE during resistance training Readiness to invest mental effort	↓ RPE ↑ Readiness to invest mental effort	[154]
Sünram-Lea et al. 2012	81 firefighter trainees (26 ± 10 y, 80.6 ± 17.1 kg)	Randomized, double-blind, placebo-controlled, crossover study	ED containing 40 mg caffeine and 50 g carbohydrates vs. ED containing 80 mg caffeine and 10.25 g carbohydrates vs. placebo	75 min prior to testing	Self-reported anxiety and stress	↓ Anxiety (in 40 mg caffeine ED) ↓ Stress (in 40 mg caffeine ED) ↔ Word recall, grammatical reasoning task, information processing, attention, fire-fighting knowledge	[148]
Schubert et al. 2013	6 male runners (22.5 ± 1.8 y; 65.4 ± 10.0 kg)	Randomized, double-blind, placebo-controlled, crossover study	ES (Guayakí Yerba Maté Organic Energy Shot™) containing 140 mg caffeine vs. ES (Red Bull Energy Shot™) containing 80 mg caffeine vs. placebo	50 min prior to testing	RPE during 5-km TT on a treadmill	↔ RPE	[176]
Wesnes et al. 2013	94 male and female volunteers (33.1 y)	Randomized, double-blind, placebo-controlled, crossover study	ES containing 157 mg caffeine, taurine, citicoline, malic acid, and glucuronolactone vs. placebo	Tests conducted hourly for 6 h following ingestion	Measures of attention Memory performance Self-reported mood outcomes	↑ Working memory ↑ Long-term memory ↑ Attentional focus ↑ Vigilance ↑ Alertness	[189]
Goel et al. 2014	15 male volleyball players (21.8 ± 6.9 y, 66 ± 7 kg)	Randomized, double-blind, placebo-controlled, crossover study	ED (Red Bull®) providing 2 mg∙kg BW^−1^ caffeine vs. placebo	60 min prior to testing	Auditory reaction time testing	↑ Auditory reaction time performance	[149]
Marczinski et al. 2014	7 male and 7 female participants (20.1 ± 2.8 y, 71.2 ± 13.1 kg)	Randomized, double-blind, placebo-controlled, crossover study	ES (5-Hour Energy®) containing 200 mg caffeine vs. placebo vs. no drink	Tests conducted 30, 90, 150, 210, 270, and 330 min following ingestion	Subjective mood outcomes Behavioral control testing (cued no-go)	↑ Feelings of vigor ↓ Fatigue ↔ Behavioral control performance	[194]
Salinero et al. 2014	90 athletes (23.9 ± 5.7 y, 70.4 ± 11.2 kg)	Randomized, double-blind, placebo-controlled, crossover study	ED (Fure®, ProEnergetics: 3 mg∙kg BW^−1^ caffeine, taurine 2000 mg, sodium bicarbonate 500 mg, L-carnitine 200 mg, and maltodextrin 705 mg) vs. placebo	60 min prior to testing	Perceived muscle power, endurance, and RPE during simulated competition	↑ Perceived muscle power during exercise	[204]
Bloomer et al. 2015	20 healthy male (n = 10; 22.7 ± 1.1 y, 82.6 ± 3.8 kg) and female participants (n = 10; 22.1 ± 0.4 y, 82.6 ± 3.8 kg	Randomized, single-blind, placebo-controlled, crossover study	ES containing 200 mg caffeine and a placebo pill vs. placebo shot with 200 mg caffeine pill vs. placebo shot and placebo capsule	Tests conducted 1, 3, and 5 h following ingestion	Cognitive performance testing Subjective measurements of energy and mood	↔ Cognitive performance ↔ Mood	[196]
Mumford et al. 2015	12 male golfers (34.8 ± 13.9 y, 81.2 ± 13.1 kg)	Randomized, double-blind, placebo-controlled, crossover study	ES (containing 155 mg caffeine, vitamin B complex, citric acid, elevATP [VDF FutureCeuticals Inc., Momence, IL] and sucralose) vs. 155 mg caffeine only vs. placebo	Provided at start and after 9^th^ hole of golf	Perceived energy and fatigue during an 18-hole round of golf	↑ Perceived energy ↓ Fatigue	[205]
Quinlivan et al. 2015	11 trained male cyclists (31.7 ± 5.9 y, 82.3 ± 6.1 kg)	Randomized, double-blind, placebo-controlled, crossover study	ED (Red Bull®) containing 3 mg∙kg BW^−1^ caffeine vs. capsule containing 3 mg∙kg BW^−1^ anhydrous caffeine vs. placebo	90 min prior to testing	RPE during TT equivalent to 1 h cycling at 75% peak power output	↔ RPE	[168]
Magrini et al. 2016	31 healthy males (n = 23) females (n = 8) (~22 y)	Randomized, double-blind, placebo controlled, parallel design	ED containing 158 mg caffeine and a blend of L-Leucine, B-Phenlyethylamine HCL, L-Valine, L-Isoleucine, N-Acetyl-L-Tyrosine, Yohimbe, Toothed Clubmoss, Yerba Mate Extract, Green Tea Extract, 5-HTP, Vinpocetine vs. placebo	30 min prior to testing	RPE during push up RTF	↔ RPE	[156]
Prins et al. 2016	18 recreational endurance runners (13 M, 5 F; 20.4 ± 3.3 y; 71.3 ± 17.2 kg)	Randomized, double-blind, placebo-controlled, crossover study	500 ml of ED (Red Bull®: 2.0 g taurine, 1.2 g glucuronolactone, 160 mg caffeine, 54 g carbohydrates (sucrose and glucose), 40 mg niacin, 10 mg pantothenic acid, 10 mg vitamin B6, and 10 mg vitamin B12) vs. placebo	60 min prior to testing	RPE during 5-km TT on a treadmill	↔ RPE	[167]
Concerto et al. 2017	14 healthy males and females (31.2 ± 9 y)	Randomized, double-blind, placebo-controlled, crossover study	ED (sugar-free Red Bull®: Aspartame 0.01 mg·kg^−1^, Caffeine 2.0 mg·kg^−1^, Taurine 25 mg·kg^−1^, glucuronolactone 15 mg·kg^−1^, niacin 0.45 mg·kg^−1^, pantothenic acid 0.15 mg·kg^−1^, vitamin B6 0.05 mg·kg^−1^, riboflavin 0.04 mg·kg^−1^, vitamin B12 0.025 mg·kg^−1^) vs. placebo	45 min prior to testing	Reaction time testing	↑ Reaction time performance	[199]
Peveler et al. 2017	15 recreational male (n = 12, 21.8 ± 2.2 y, 84.0 ± 15.4 kg) and female (n = 3, 24 ± 4.4 y, 72.6 ± 6.6 kg) runners	Randomized, double-blind, placebo-controlled, crossover study	ED (Red Bull®: 248.42 mL, 80 mg of caffeine, 1,000 mg of taurine) vs. ED (Monster®: 473.18 mL, 163 mg of caffeine, 1,000 mg of taurine,) vs. ES (5-Hour Energy®: 27.5 mL, 207 mg of caffeine, 479.9 mg of taurine) vs. placebo	60 min prior to testing	RPE during 15 minutes running at 70% VO_2_max	↔ RPE	[183]
Wesnes et al. 2017	24 healthy male and female volunteers (22.1 ± 5.3 y, 64.2 ± 12.6 kg)	Randomized, double-blind, placebo-controlled, crossover study	ES (B-vitamins Vitamin B6 – 40 mg, Niacin (Vitamin B3) – 30 mg, Vitamin B12 – 500 μg, Folic acid (Vitamin B9) 400 μg, Energy blend [1870 mg total: Taurine, Glucuronolactone (glucuronic acid), Malic acid, N-acetyl-L-tyrosine, L-phenylalanine, Caffeine 157 mg, Citicoline) vs. placebo	Tests conducted 30, 60, and 90 min following ingestion.	Cognitive function tests Self-reported mood and alertness	↑ Working memory ↑ Episodic memory ↑ Attention ↑ Reaction time	[190]
Chtorou et al. 2019	19 male physical education students (21.2 ± 1.2 y; 76.6 ± 12.6 kg)	Randomized, double-blind, placebo-controlled, crossover study	ED (Red Bull®) containing 160 mg caffeine, 2.0 g taurine, 1.2 g glucuronolactone, 54 g carbohydrate, 40 mg niacin, 10 mg pantothenic acid, 10 mg vitamin B6, and 10 μg vitamin B12) vs. placebo	60 min prior to testing	Visual reaction time testing Subjective mood outcomes	↑ Reaction time performance ↓ Measures of confusion ↓ Measures of fatigue ↓ Measures of anger ↓ Measures of anxiety ↑ Measures of vigor ↓ RPE during testing	[146]
Thomas et al. 2019	9 elite League of Legends esports players (21 ± 2 y, 25.6 ± 3.4 kg)	Randomized, double-blind, placebo-controlled, crossover study	ED (AI Reload®: 12 g Glycerin, 150 μg Vitamin B12, 70 mg Magnesium, 40 mg Sodium, and 1005 mg Proprietary Blend [L-Carnitine, 150 mg Caffeine, L-theanine, Phosphatidylserine, Choline [from alpha-GPC], Nicotinamide Adenine Dinucleotide (reduced form NADH)] vs. placebo	30 min prior to testing	Measures of attention Reaction time testing Working memory tests	↔ Attention ↔ Reaction time ↑ Working memory	[188]
Garcia-Alvarez et al. 2020	223 healthy male and female volunteers (18-70 y)	Randomized, double-blind, placebo-controlled, crossover study	ES (5 Hour Energy®: Vitamin B6 (pyridoxine hydrochloride) 40 mg, Folic acid 400 μg, Vitamin B12 (cyanocobalamin) 500 μg, Sodium 18 mg, Energy blend [2009 mg Total of: Taurine, choline, glucuronic acid (as or from glucuronolactone), N-acetyl l-tyrosine, l-phenylalanine, and malic acid, Caffeine 6 mg, other ingredients: Purified water, natural and artificial flavors, sucralose, Decaf: containing no caffeine) vs. placebo	Tests conducted 0.5, 2.5, and 5 h following ingestion.	Reaction time and accuracy tests Distraction avoidance test Measures of mood	↔ Reaction time performance ↔ Distraction avoidance ↔ Mood	[197]
Reis et al. 2021	12 males (22 ± 2.6 y, 74.4 ± 5.5 kg)	Randomized, double-blind, placebo-controlled, crossover study	ED (123 kcals, 3 mg∙kg BW^−1^ caffeine, 30 g of carbohydrates, 24 mg of sodium, 1,000 mg of taurine) vs. sugar-free ED (12 kcals, 3 mg∙kg BW^−1^ caffeine, 0 g of carbohydrates, 24 mg of sodium, 1,000 mg of taurine) vs. placebo	40 min prior to testing	RPE during 55-minute treadmill run at 65-75% VO_2_max	↓ RPE	[185]

↑ = ED/ES significantly greater (p < 0.05) than control; ↓ = ED/ES significantly less (p < 0.05) than control; ↔ = no significant difference between ED/ES and control; ED = energy drink; ES = energy shot; g = gram; h = hours; kg = kilogram; km = kilometer; mg = milligram; min = minutes; RPE = rating of perceived exertion; RTF = repetitions to fatigue; TT = time trial; TTE = time to exhaustion; y = years; μg = microgram

**Table 9.** Sport-Specific Performance Outcomes in Acute Energy Drink and Energy Shot Studies.

Study	Subjects	Design	ED/ES Condition	Timing	Performance Testing Protocol	Results	Ref #
Del Coso et al. 2013b	26 elite male rugby players (25 ± 2 y, 93 ± 15 kg)	Randomized, double-blind, placebo-controlled, crossover study	ED (Fure®, ProEnergetics at 1 and 3 mg∙kg BW^−1^ caffeine, taurine 2000 mg, sodium bicarbonate 500 mg, L-carnitine 200 mg, and maltodextrin 705 mg) vs. placebo	60 min prior to testing	Body impacts during simulated rugby game	↑ Body impacts	[169]
Peltier et al. 2013	8 trained male tennis players (26.0 ± 5.7 y; 82 ± 11 kg	Randomized, double-blind, placebo-controlled, crossover study	ED (caffeine; 140 mgL^−1^) vs. placebo	Consumed before, during, and after tennis matches	Two 3-match round robin tennis tournaments	↑ Tennis stroke frequency	[209]
Del Coso et al. 2014	15 male volleyball players (21.8 ± 6.9 y, 66 ± 7 kg)	Randomized, double-blind, placebo-controlled, crossover study	ED (Fure®, ProEnergetics at 1 and 3 mg∙kg BW^−1^ caffeine, taurine 2000 mg, sodium bicarbonate 500 mg, L-carnitine 200 mg, and maltodextrin 705 mg) vs. placebo	60 min prior to testing	Ball spike test Agility T-test Simulated volleyball match	↑ Ball spike velocity (75 ± 10 vs. 73 ± 9 km/h) ↓ T-test time (10.8 ± 0.7 vs. 10.3 ± 0.4 s) ↑ Frequency of successful volleyball actions (34.3 ± 16.5 vs. 24.6 ± 14.3%)	[136]
Monaghan et al. 2014	10 active-duty police officers	Randomized, single-blind, placebo-controlled, crossover study	ES (5-Hour Energy Extra®) vs. placebo	30 min prior to testing	Handgun shooting stability test	↓ Handgun stability	[210]
Mumford et al. 2015	12 male golfers (34.8 ± 13.9 y, 81.2 ± 13.1 kg)	Randomized, double-blind, placebo-controlled, crossover study	ES (containing 155 mg caffeine, vitamin B complex, citric acid, elevATP [VDF FutureCeuticals Inc., Momence, IL] and sucralose) vs. 155 mg caffeine only vs. placebo	Provided at start and after 9^th^ hole of golf	18-hole round of golf	↑ Total score (76.9 ± 8.1 vs. 79.4 ± 9.1) ↑ Drive distance (239.9 ± 33.8 vs. 233.2 ± 32.4 m) ↑ Greens in regulation (8.6 ± 3.3 vs 6.9 ± 4.6)	[205]
Perez-Lopez et al. 2015	13 elite female volleyball players (25.2 ± 4.8 y, 64.4 ± 7.6 kg)	Randomized, double-blind, placebo-controlled, crossover study	ED (Fure®, ProEnergetics at 1 and 3 mg∙kg BW^−1^ caffeine, taurine 2000 mg, sodium bicarbonate 500 mg, L-carnitine 200 mg, and maltodextrin 705 mg) vs. placebo	60 min prior to testing	Standing ball spike test Jumping spike test Agility T-test Simulated volleyball match	↑ Standing ball spike velocity (19.7 ± 1.9 vs. 19.2 ± 2.1 m/s) ↑ Jumping spike velocity (18.8 ± 2.2 vs. 17.9 ± 2.2 m/s) ↓ T-test time (10.9 ± 0.3 vs. 11.1 ± 0.5 s) ↑ Frequency of successful volleyball actions (34 ± 9 vs 14 ± 9%)	[140]
Clarke et al. 2016	12 male badminton players (28 ± 9 y, 78 ± 9 kg)	Randomized, double-blind, placebo-controlled, crossover study	Drink providing 4 mg/kg caffeine vs. drink providing 4 mg∙kg BW^−1^ caffeine and 6.4% carbohydrates vs. placebo	60 min prior to testing	Badminton serve accuracy test	↑ Short serve accuracy ↑ Long serve accuracy	[201]
Portillo et al. 2017	16 elite female rugby sevens players (23 ± 2 y, 66 ± 7 kg)	Randomized, double-blind, placebo-controlled, crossover study	ED (Fure®, ProEnergetics at 1 and 3 mg∙kg BW^−1^ caffeine, taurine 2000 mg, sodium bicarbonate 500 mg, L-carnitine 200 mg, and maltodextrin 705 mg) vs. placebo	60 min prior to testing	Body impacts during three rugby sevens matches	↑ Body impacts	[208]

↑ = ED/ES significantly greater (p < 0.05) than control; ↓ = ED/ES significantly less (p < 0.05) than control; ↔ = no significant difference between ED/ES and control; ED = energy drink; ES = energy shot; g = gram; kg = kilogram; km = kilometer; mg = milligram; min = minutes; y = years

## 5. Force and power production

Though the acute effects of ED and ES consumption on force production and power production have been well-studied, substantial variability exists between the results of similar investigations (Table 4). For example, of the 13 investigations examining some aspect of lower-body power production, nine reported beneficial effects of ED/ES consumption [133-141], while four did not [142-145]. It is possible that these differences may be partially driven by differing caffeine dosages used in the study protocols, or that those receiving the intervention were not equal across all of the studies (exercise naïve versus exercise-trained), in addition to the genetic profile of the individuals, which may influence the rate of caffeine metabolism [129]. Furthermore, variability in caffeine content and supplemental ingredients may also influence the likelihood of an ergogenic benefit following ED/ES consumption. In a series of closely-related investigations, the research group of Del Coso and colleagues demonstrated that acute consumption of 3 mg∙kg of bodyweight (BW)^−1^ caffeine from energy drinks resulted in increased countermovement jump height in male semiprofessional soccer players [135], college-aged female soccer players [138], and male sprint swimmers [139]. They also reported greater power output during a 15-s multiple jump series in elite female rugby sevens players [137], greater power output during bench press and half-squat power-load testing [141], as well as increased jump height in male college volleyball players [136] and elite female volleyball players [140]. These results were mirrored by several other investigations by the same group, which found improvements in jump height in adolescent basketball players [134] and increased jump height and peak power in elite badminton players [133]. In contrast, Kammerer et al. [145] found no effect of an ED containing 80 mg caffeine (approx. 1.2 mg∙kg BW^−1^) on vertical jump performance in Colombian army soldiers. Similarly, Campbell et al. [142] provided a ES containing 2.5 mg∙kg BW^−1^ caffeine to college-aged males and females, with no demonstrable effect on jumping performance. Jacobson and colleagues [144] likewise did not identify any between-group differences in jump performance following the ingestion of an ES containing approximately 3.12 mg∙kg BW^−1^ caffeine in college-aged males and females. Based on these findings, it appears that ED and ES containing at least 3 mg∙kg BW^−1^ caffeine are most likely to benefit maximal lower-body power production, which is consistent with the current consensus in caffeine research [13]. Furthermore, it is worth noting that the majority of studies which tended to report positive findings regarding the ergogenic potential of ED/ES were conducted in athletes.

To date, a variety of investigations examining the effects of ED/ES consumption on maximal force production have shown beneficial results, though outcomes vary depending on the exercise model employed. These studies included assessments of handgrip maximal voluntary contraction (MVC) force [133,134,139,143,145-150], bench press [151] and leg press [152] 1RM testing, as well as other lower-body maximal isometric and dynamic contraction tests [152]. In general, it appears that ED/ES ingestion has a positive effect on handgrip MVC force, though null findings have also been reported. Astley and colleagues [150] reported that handgrip MVC was improved following ED consumption (containing 2.5 mg∙kg BW^−1^ caffeine) in resistance-trained males, findings which were in accordance with a similar study by Chtourou et al. [146] which recruited active, college-aged male participants as well as several other investigations in firefighters [148], sprint swimmers [139], and elite junior tennis players [147]. Interestingly, the study which recruited sprint swimmers found that an ED containing 3 mg∙kg BW^−1^ caffeine improved right-hand but not left-hand gripping performance, suggesting the presence of a more nuanced ergogenic effect perhaps related to neural coordination [139]. Similarly, several studies found no effect of ED/ES consumption on handgrip performance in college-aged males and females [149], elite badminton players [133], and Colombian army soldiers [145], though as mentioned previously, the latter study used a ED containing only 80 mg caffeine (approximately 1.2 mg∙kg BW^−1^).

In contrast to the previous studies examining handgrip performance, ED/ES have not been conclusively shown to benefit maximal force production in dynamic movements using larger muscle groups such as bench press or leg press. For example, Eckerson et al. [151] recruited 17 resistance-trained, college-aged males to consume an ED containing 160 mg caffeine (approx. 1.87 mg∙kg BW^−1^) prior to bench press 1RM testing. The researchers found no effect of ED on 1RM, which could potentially be due to the comparatively low relative dose of caffeine contained in the ED product. Similarly, Harty and colleagues [152] recruited 16 resistance-trained males and females to examine the ergogenic potential of a ED containing 300 mg caffeine (approx. 4.1 mg∙kg BW^−1^) and 20 g protein. The participants completed a lower body testing battery consisting of maximal isometric and isokinetic strength testing using a specialized squat dynamometer as well as leg press 1RM testing. Despite the relatively higher caffeine content of the ED compared to many energy products, the researchers reported null findings for the performance outcomes. In summary, though more information is needed in this area, ED/ES do not appear to reliably enhance maximal strength in multi-joint movements.

## 6. Muscular endurance

It appears that ED/ES consumption may improve muscular endurance in some populations, though results are mixed, as summarized in Table 5. Forbes and colleagues [153] provided active male and female participants with an ED containing 2 mg∙kg BW^−1^ caffeine prior to muscular endurance testing, which consisted of bench press repetitions to fatigue at 70% 1RM. The ED condition significantly increased the total number of repetitions completed. Likewise, Duncan and colleagues [154] recruited resistance-trained males to complete a testing bout consisting of one set each of bench press, deadlifts, chest-supported rows, and back squats, all completed to volitional fatigue at 60% 1RM. In a crossover fashion, the researchers administered either ES (containing 179 mg caffeine) or placebo 60 minutes prior to exercise and found that consumption of the ES resulted in significantly greater repetitions to fatigue completed across all exercises. Dawes and colleagues [155] likewise reported pre-exercise consumption of an ED resulted in a significant increase in pushups completed to fatigue (approximately 12.2%, compared to a 3.3% increase after consumption of placebo). In contrast, several investigations have found minimal effects of ED/ES on muscular endurance. For example, Eckerson and colleagues [151] administered an ED containing approx. 1.87 mg∙kg BW^−1^ caffeine to physically active male participants prior to completing bench press repetitions to fatigue at 70% 1RM. The researchers found no between-group differences in either the ED condition, a caffeine-matched control, or placebo. Similarly, Campbell et al. [142] reported that an ES containing approx. 2.4 mg∙kg BW^−1^ caffeine provided no ergogenic benefits to local muscular endurance assessed via YMCA bench press test and curl-up test. Magrini and colleagues [156] as well as Harty and colleagues [152] likewise found no effect of ED consumption on measures of muscular endurance, assessed via pushups and submaximal leg press repetitions to fatigue, respectively.

## 7. Anaerobic capacity

The ergogenic potential of ED/ES to improve anaerobic capacity has been extensively studied, with largely null findings reported. Many of these investigations assessed lower-body anaerobic performance via the Wingate anaerobic cycle test [153,157-159]. As stated above, Forbes et al. [153] administered an ED with 2 mg∙kg BW^−1^ caffeine to active, college-aged participants prior to performance testing, which included three repeated 30 second (s) Wingate cycle tests. The drink did not improve peak or average power production across the tests. Similarly, Campbell and colleagues [157] recruited 15 recreationally-active males and females to complete two 20s Wingate test after consumption of an ED containing 160 mg caffeine (approx. 2.1 mg∙kg BW^−1^), finding no effect of the beverage on performance outcomes. Hoffman and colleagues [158] also assessed Wingate performance in male strength and power athletes, reporting that consumption of an ED had no demonstrable effect on anaerobic performance. These findings were echoed by those of Zileli et al. [159], who found no effect of ED ingestion on Wingate performance in amateur athletes. These findings may be explained by the relatively low dose of caffeine employed (80 mg, approx. 1.1 mg∙kg BW^−1^). Several investigations have also assessed the effects of ED/ES on sprinting performance in the laboratory context [142,160-162]. Astorino and colleagues [160] administered an ED containing approximately 1.3 mg∙kg BW^−1^ caffeine to female collegiate soccer players prior to repeated sprint testing, with no demonstrable effect on performance. Campbell et al. [142] likewise found no effect of an ED containing approximately 2.4 mg∙kg BW^−1^ caffeine on sprint speed during repeated sprint testing. Of note, Alford and colleagues [161] reported that the consumption of an ED containing only 80 mg caffeine resulted in significant improvements in anaerobic capacity, as measured by how long subjects were able to maintain maximal cycling speeds without decreasing. It is possible that multi-ingredient ED/ES products may contain ingredients that act against the known effects of caffeine, thus yielding conflicting results. In summary, it appears that ED/ES products providing less than 3 mg∙kg BW^−1^ caffeine confer minimal ergogenic benefits on anaerobic capacity.

## 8. Aerobic exercise

The impact of ED and ES on aerobic performance has received considerable attention. Though the results have been mostly positive, there are also a number of studies which have found no effect, as summarized in Table 6. Several investigations have suggested these products may improve time-to-exhaustion during extended aerobic exercise [163] and during treadmill aerobic testing protocols [164,165], as well as benefit time-trial performance [166,167]. Geiß and colleagues [163] recruited ten endurance athletes who consumed an ED containing 160 mg caffeine (approx. 2 mg∙kg BW^−1^) and 54 g carbohydrate prior to cycling exercise, which culminated with a time-to-exhaustion ramp protocol. The researchers reported that the ED resulted in significantly longer time-to-exhaustion relative to placebo. Similarly, Prins et al. [167] found that the consumption of the same ED formulation reduced the time to complete a 5-km run in a mixed-sex cohort of recreational endurance athletes. These results were also mirrored in a cycling model by Ivy and colleagues [166], who provided the same ED product to trained male and female cyclists prior to a standardized time trial (equivalent to the work required to complete 1 h of cycling at 70% maximal wattage). The researchers found that the cyclists completed the time trial approximately 4.7% faster after consuming the ED compared to the placebo trial. Alford et al. [161] also investigated the ergogenic potential of the same ED formulation in a 2001 study, though using half the serving size of the aforementioned investigations. The researchers recruited college-aged males and females to perform cycling exercise at 65-75% maximum heart rate until their heart rate exceeded the upper threshold of 75%, finding that cycle time was enhanced by ED consumption compared to plain water and carbonated water placebo conditions. Positive findings have also been reported by Quinlivan and colleagues [168], who administered the same product formulation in a bodyweight-dependent relative dose to provide 3 mg∙kg BW^−1^ caffeine to trained male cyclists. The researchers reported performance improvements of approximately 2.8% during an extended cycling time trial. Several studies have also examined the utility of ED/ES to improve aerobic performance during exercise testing protocols, with promising results. Kazemi and colleagues [164] recruited college-aged females to perform a Bruce treadmill test following the consumption of 6 ml∙kg BW^−1^ of two ED formulations, both of which increased time-to-exhaustion during the test compared to placebo. Similar findings were also reported by Rahnama and colleagues [165], who administered ED formulations to college-aged males prior to a Bruce treadmill testing protocol.

The potential of ED to improve aerobic exercise performance in the team sport context has also been investigated, again with largely positive results. As mentioned above in the section on power production, the research group of Del Coso and colleagues performed a series of investigations examining the ergogenic potential of ED formulations providing 3 mg∙kg BW^−1^ caffeine on various aspects of sport performance. The researchers recruited a wide variety of athletes, finding that consumption of an ED resulted in a greater total distance covered at a speed greater than 13 km∙h^−1^ during a simulated soccer match in semiprofessional soccer players [135], greater running speed during a rugby sevens competition in elite female rugby athletes [137], increased total distance covered during a simulated competition in elite male rugby athletes [169] and college-aged female soccer athletes [138], as well as increased distance covered while running at high intensity during a simulated competition in male field hockey players [170]. Additionally, the researchers found that ED consumption resulted in a faster high intensity running pace during a tennis match in elite junior tennis players compared to placebo [147].

In contrast, several investigations have reported null findings when examining the effects of ED on endurance exercise performance [145,159,168,171-176]. Candow and colleagues [173] provided college-aged males and females with a sugar-free ED containing 2 mg∙kg BW^−1^ caffeine prior to a time-to-exhaustion running protocol at 80% VO_2_max, finding no ergogenic benefit of the ED. Similar findings were reported by Nelson and colleagues [174], who found no effect of an ED containing 2 mg∙kg BW^−1^ caffeine on cycling time-to-exhaustion at 100% ventilatory threshold. Studies employing a time-trial model have also shown null results, as reported by Philips et al. [175], who recruited cyclists to complete a 25-km simulated road race after consumption of an ED (160 mg caffeine, 2 g taurine, 1.2 g glucuronolactone, 56 g carbohydrate, and B vitamins) versus a cola product matched for caffeine and carbohydrate and compared to a flavored sparkling water placebo; Schubert et al. [176], who assessed the impact of two ES formulations ES (Guayakí Yerba Maté Organic Energy Shot™, containing 140 mg caffeine) versus ES (Red Bull Energy Shot™, containing 80 mg caffeine) compared to a placebo on 5-km running performance, and Alansare et al. [172], who examined the ergogenic potential of an ED (240 mL of 8% calamansi juice, 10% glucose, 0.8% taurine, and 0.4% BCAA) on 3-km running performance. Several studies have also reported null findings when examining the impact of ED formulations on time-to-exhaustion during standardized aerobic exercise testing protocols such as the Bruce treadmill test [145,171]. In summary, it appears that ED/ES (particularly formulas that contain at least 2 mg∙kg BW^−1^ caffeine) have the potential to improve aerobic performance, though findings are mixed.

## 9. Metabolic effects/fuel utilization

In general, it appears that ED and ES consumption have the potential to increase acute measures of resting metabolism, which could theoretically result in preferential changes in body composition over time with repeated use (Table 7). However, it is important to note that few studies in this area have assessed metabolic outcomes after consumption of an ED or ES but instead utilized various ready-to-drink thermogenic products [177-182] which are purported to acutely increase fat metabolism and energy expenditure. Like ED/ES, these products contain caffeine, but generally have dosages much higher than typical energy products [1,2]. Thermogenic products often include a variety of other ingredients such as epigallocatechin gallate (EGCG, a naturally occurring polyphenol in green tea), carnitine, and conjugated linoleic acid [131,179]. Based on the current body of literature in this area, it appears that the acute consumption of caffeine-containing thermogenic products results in greater energy expenditure and lipolysis at rest [177-182]. A recent study [152] using an ED formulation containing 300 mg caffeine (approx. 4.1 mg∙kg BW^−1^) and 20 g of protein likewise reported acute increases in resting metabolism within 60 minutes of consumption and following a standardized resistance exercise protocol [152]. However, it is important to note that the ED and placebo conditions were not calorie-matched, as the placebo contained approximately 100 kcal less than the supplement condition. Conversely, an earlier investigation by Del Coso and colleagues [141] found no effect of ED formulations delivering 1 or 3 mg∙kg BW^−1^ caffeine on measures of resting metabolism. Importantly, acute increases in lipolysis and beta-oxidation may not equate to meaningful reductions in body weight or fat mass over time; therefore, research is warranted in order to draw more definitive conclusions in regard to the utility of ED/ES as part of a weight management plan.

Available research suggests that energy products have little impact on fuel utilization during exercise, though information in this area is limited. For example, Peveler and colleagues [183] examined the impact of two caffeine and carbohydrate-containing ED formulations and one ES formulation on running economy in a mixed-sex cohort of recreational runners. After determining each subjects’ maximal oxygen uptake (VO_2_max), the researchers administered the supplemental and placebo condition in crossover fashion prior to 15 minutes of running at 70% VO_2_max, finding no effect of the energy products on running economy compared to placebo. Kinsinger and colleagues [184] likewise examined the effect of ES ingestion (containing approx. 100 mg caffeine) on oxygen consumption and substrate utilization during a Bruce treadmill exercise test, finding no effect of the supplement on respiratory exchange ratio (RER) or VO_2_peak. In contrast, Reis et al. [185] administered a carbohydrate and caffeine-containing ED and a sugar-free formulation (both containing 3 mg∙kg BW^−1^ caffeine) to college-aged males prior to an endurance exercise protocol. Interestingly, the researchers reported decreases in RER during the first five minutes of exercise and after 40-45 minutes of exercise following consumption of the sugar-free but not the carbohydrate-containing formulation. Clearly, more research is required in this area.

## 10. Cognitive effects

ED and ES appear to generally have a positive effect on aspects of cognitive function such as memory [148,161,186-191], as summarized in Table 8. Early research by Alford and colleagues [161] found that acute consumption of an ED containing 80 mg caffeine resulted in significant improvements in measures of memory and concentration during cognitive testing. Similarly, Scholey et al. [187] administered an ED containing 75 mg caffeine prior to a cognitive testing battery. Of the five aspects of cognitive performance assessed, only two (‘secondary memory’ and ‘speed of attention’) were positively affected by consumption of the beverage. Sünram-Lea and colleagues [148] likewise reported positive effects of ED ingestion on memory performance in firefighter trainees after consumption of a formulation containing 50 g glucose and 40 mg caffeine (approx. 0.5 mg∙kg BW^−1^). Interestingly, Wesnes et al. [190] found improvements in working and episodic memory following consumption of a caffeinated, carbohydrate-containing ED formulation but not an identical sugar-free version. However, several studies have shown improvements in memory performance after the consumption of sugar-free energy shots. Thomas and colleagues [188] provided an ES containing 150 mg caffeine (approximately 1.9 mg∙kg BW^−1^) to nine elite esports players prior to a cognitive testing battery. Though null effects were found for most outcomes, the ES significantly improved performance in the n-back test, a measure of working memory. Wesnes et al. [189] also administered an ES containing 157 mg caffeine to adult male and female subjects, finding positive effects of the product on measures of attentional focus, concentration, and vigilance as well as working and long-term memory. Based on the results of studies examining ED and ES ingredients in isolation, it appears that the caffeine content of these products is likely the primary mechanism of action for such cognitive benefits, though carbohydrate may also play a role [187,192,193]. However, it should be noted that several studies have found no effect of caffeine-containing [145,194-196] as well as caffeine-free [197] energy products on various measures of cognitive and memory performance. Moreover, positive effects of ED and ES consumption may be more pronounced during times of fatigue or situations of duress such as sleep deprivation and exercise-induced exhaustion.

## 11. Reaction time

Acute ingestion of caffeine-containing ED and ES has also been shown to improve reaction time performance in a variety of populations. In a 2009 study, Hoffman and colleagues [158] demonstrated the positive effects of ED ingestion on reaction time in male strength and power athletes. The researchers provided either placebo or a caffeinated ED prior to reaction time testing, which consisted of auditory and visual stimuli that would prompt the subjects to lunge forward and contact the testing device. Ultimately, ED ingestion improved the total number of successful contacts and total percentage of successful contacts completed during testing. Alford et al. [161] likewise found that choice reaction time was improved following ingestion of an ED containing 80 mg of caffeine, in addition to taurine and carbohydrate; all of which may have influenced choice reaction time. These results were further supported by Goel and colleagues [149], who provided the same formulation to college-aged medical students and found that auditory reaction time was significantly improved relative to control. Howard et al. [198] also assessed reaction time using the same product formulation, though three bodyweight-dependent doses were provided to participants that were the equivalent of a half serving of the beverage (1.8 ml ED∙kg BW^−1^), a single serving (3.6 ml ED∙kg BW^−1^) or 1.5 servings (5.4 ml ED∙kg BW^−1^) of the beverage. Interestingly, the researchers found that the smallest dose resulted in the greatest improvements in reaction time performance compared to placebo, though the other two doses were still beneficial. A study conducted by Concerto and colleagues [199] also found similar results using a sugar-free formulation of the same product, which was further supported by two studies that utilized ED ingredients in isolation [200,201]. In a more recent study, Evans et al. [202] found improvements in Pattern Comparison Processing Speed as well as a lower number of false starts during a Psychomotor Vigilance Test following acute consumption of an ED containing 300 mg of caffeine. However, not all studies have reported positive effects of ED/ES ingestion on reaction time performance, as several studies reported null findings when assessing an ES formulation containing approximately 200 mg caffeine [194,195]. A study by Antonio et al. [203] also reported null findings in regards to reaction time 30-min after consumption of an ED containing 300 mg of caffeine in exercise-trained men and women. However, the authors [203] did observe a an improvement in psychomotor vigilance mean reaction time compared to the Placebo condition (ED 473.8 ± 42.0 milliseconds vs. PL 482.4 ± 54.0 milliseconds; p = 0.0220).

## 12. Subjective effects

ED and ES have been consistently shown to improve subjective outcomes such as mood, alertness, and feelings of energy and focus. However, these products seem to have a less consistent effect on rating of perceived exertion (RPE) during exercise. Chtourou and colleagues [146] demonstrated that consumption of an ED containing approximately 1 mg∙kg BW^−1^ caffeine could positively affect aspects of mood, finding significant reductions in self-reported measures of confusion, fatigue, anger, and anxiety as well as significant improvements in self-reported vigor. Seidl et al. [200] also investigated the same product formulation in college-aged graduate students, reporting that feelings of well-being, vitality, and social extraversion were maintained after consumption of the ED but not placebo. Duncan et al. [154] likewise noted that athletes who consumed an ED reported significantly greater readiness to invest mental effort. Salinero and colleagues [204] recruited 90 experienced athletes and found that consumption of an ED containing 3 mg∙kg BW^−1^ caffeine increased the athletes’ self-perceived muscular power production during exercise testing. Interestingly, energy products may also reduce negative subjective states associated with stressful situations. Sünram-Lea et al. [148] found that participants who consumed an ED containing 40 mg caffeine and 50 g glucose (approx. 0.5 mg caffeine∙kg BW^−1^) reported significantly lower levels of anxiety and stress during a three-day firefighter training class compared to those who consumed placebo. It should be noted, however, that several investigations reported null findings on mood following ingestion of caffeine-containing [196] and caffeine-free [197] energy products.

Unsurprisingly, caffeine-containing energy products have consistently been shown to improve measures of energy, vigor, focus, and alertness in a variety of populations. Hoffman and colleagues [158] provided a caffeinated ED to male strength and power athletes, finding that the supplement condition resulted in significant improvements in subjective feelings of focus and energy. Alford et al. [161] likewise administered an ED containing 80 mg caffeine to subjects prior to cognitive and physical tests, reporting that the subjects felt significantly increased subjective alertness. This benefit was also observed by Wesnes and colleagues [189], who found that sleep-deprived subjects reported increased alertness after ingestion of a caffeine-containing, sugar-free energy shot. Several studies have likewise found that energy products can reduce subjective ratings of fatigue during repeated testing [194,198,205]. Of note, Mumford and colleagues [205] provided golfers with a caffeine-containing ES during an 18-hole round of golf, finding that the golfers reported more energy and less fatigue over the round following ingestion of the ES compared to placebo. However, it should be noted that unlike these demonstrated reductions in subjective fatigue, it appears that ED/ES have a less consistent effect on perceived exertion during exercise. In this respect, several investigations [146,154,185,206] found that RPE during exercise was lower after ED/ES ingestion, while several others have reported no difference [156,167,168,173,176,183,207].

## 13. Sport-specific performance

As presented in Table 9, several investigations have demonstrated the effectiveness of ED/ES to improve sport-specific performance in a variety of athletic contexts. Del Coso and colleagues [169] administered an energy drink containing 3 mg∙kg BW^−1^ caffeine to elite rugby players prior to a simulated match and found that athletes who ingested the ED had significantly greater body impacts during the game. These findings were mirrored by a later investigation by the same group [208], which examined game performance of elite female rugby sevens athletes after ED ingestion. As before, the athletes who consumed the ED had significantly more body impacts at a variety of collision intensities compared to those who had placebo, suggesting that the players were more engaged during the event after ED ingestion. Del Coso and colleagues [136] also examined sport-specific performance in male volleyball players after ingestion of an ED containing 3 mg∙kg BW^−1^ caffeine and found significant improvements in maximal ball velocity during a volleyball spike test, greater agility performance, and a greater frequency of successful volleyball actions during a simulated game in the ED condition compared to placebo. The same research group [140] also conducted a similar study in elite female volleyball players, again finding that ED ingestion increased ball velocity during the spike test, improved agility performance, and improved the frequency of successful volleyball actions during a simulated volleyball game. ED/ES have also been shown to improve performance in racquet sports, as shown by Peltier et al. [209] in a 2013 study on tennis performance. Over the course of a three-match round-robin tennis tournament, well-trained male tennis players ingested a caffeine containing ED (total dose approximately 2.2 mg∙kg BW^−1^) or placebo. The researchers found that stroke frequency across the tournament was significantly greater in the ED condition compared to placebo, suggesting that the athletes played with more intensity after ED ingestion. Clarke and colleagues [201] likewise demonstrated that ED ingredients such as carbohydrates and caffeine could improve performance in badminton players, finding that a solution containing carbohydrates and 4 mg∙kg BW^−1^ caffeine improved short serve and long serve accuracy during sport-specific tests. ES ingestion, in combination with B vitamins and polyphenols, has also been shown to greatly improve golf performance in trained golfers, with significant improvements in drive distance and reductions in total score across 18 holes [205]. However, the use of energy products may prove detrimental in shooting sports, as shown in an unpublished study by Monaghan et al. [210]. The researchers recruited ten active police officers to perform aiming exercises using a mock handgun testing device 30 minutes after ingestion of an ES containing 230 mg caffeine. They found that aiming stability was significantly impaired in the ES but not placebo condition, though the impact on actual shooting accuracy was not determined. Based on these results, ED and ES might need to be avoided prior to marksmanship events that require aiming precision.

## 14. Sex differences

The potential for differences between genders to impact metabolism, substrate oxidation, and performance outcomes related to ED/ES use is an important area that is largely unexplored. In this respect, it is well-established that strength [211,212], power production [213], and neuromuscular function [214] is dependent upon menstrual cycle phase with the divergent circulating patterns of estrogen and progesterone being key mechanistic factors to explain these differences. Beyond exercise performance, menstrual cycle phase also impacts oxidation rates of carbohydrate and fat [215] and can also impact the speed at which caffeine, a primary ingredient in ED/ES, is metabolized [216], with caffeine elimination being reduced during the early follicular versus other phases of the menstrual cycle [216]. Finally, oral contraceptive use has been identified as another variable which may impact physical performance, substrate oxidation, and caffeine metabolism in females [217].

To further extend the impact of these factors, recent evidence by Santana et al. [211] illustrated that the performance decrements observed during the early follicular phase may be mitigated by caffeine administration. In this study, 14 healthy, eumenorrheic women completed a series of strength, power, and endurance tests during the early follicular and mid-follicular phases of their menstrual cycle while consuming either a placebo or caffeine. As expected, strength performance was decreased during the early follicular when compared to the mid-follicular phase. However, when caffeine, a key ingredient in many ED/ES, was provided during the early follicular phase, the previously observed strength decrements were mitigated. While more research is needed to fully understand how gender may impact the metabolism of different ingredients commonly found in ED/ES, current findings clearly indicate that based upon menstrual cycle phase and oral contraceptive use that performance, substrate utilization, and caffeine metabolism can all be impacted by gender and these factors should be considered when evaluating future recommendations regarding ED/ES use.

## 15. Continued use and long-term adaptations

As demonstrated throughout, many investigations have employed various types of short-term interventions to examine the impact of an ED or ES on some measure of exercise performance, metabolism, cognition, and affect (See Tables 3 – 8). Specifically, several acute studies have reported short-term increases in resting energy expenditure [152,218-220] following ED ingestion, which could promote greater weight and/or fat loss over time when combined with an exercise program. An important caveat to this would be the energy (kcal) content of the ED beverage being consumed, as a high energy or sugar content could negate any short-term increases in energy expenditure if the goal is weight loss. While a substantial number of investigations are available that have examined short-term outcomes, only three studies appeared to have been published that examined ongoing ingestion of an ED beyond the initial day of ingestion and the subsequent effects on body composition or performance parameters. These findings are of particular interest for those people who may harbor concerns over the safety of ED consumption.

Steinke et al. [221] had 15 healthy adults consume two cans (500 mL total volume) of an ED (containing 1000 mg of taurine, 100 mg of caffeine, B5, B6, and B12 vitamins, glucuronolactone, and niacinamide) daily for one week. Blood pressure, heart rate, and electrocardiogram (ECG) parameters were then assessed before and after consuming a 500 mL dose each day for seven days. The authors reported that ED consumption increased systolic blood pressure within four hours by 8% on day 1 and 10% on day 7. Diastolic blood pressured increased within two hours of consumption, by 7% on day 1 and 8% on day 7. On day 1, heart rate increased by 8% and 11% on day 7. No clinically relevant changes in ECG parameters were observed following acute and repeated ED consumption. It is important to note these acute hemodynamic responses are similar to those reported following caffeine (alone) and coffee [222,223]; which, unless underlying comorbidities exist, namely hypertension or cardiovascular disease, are likely not clinically relevant. However, it is currently unknown if this would apply to ED/ES as well, particularly when considering the additional ingredients commonly found in these beverages.

Roberts and colleagues [224] had 60 healthy, college-aged males and females consume either a carbonated, low-calorie ED or a carbonated, commercially available diet soda for 28 days while being assessed for changes in anthropometrics, body composition, hemodynamics, resting metabolic rate, markers of lipolysis, and adverse events. Participants were randomly assigned in a single-blind fashion according to their baseline fat mass values. Each daily dose of a low-calorie ED delivered 10 kcals, 200 mg of caffeine (approximately 2.8 mg∙kg^−1^), including a proprietary blend of guarana extract (caffeine source), green tea, glucuronolactone, ginger extract, and taurine. Participants assigned to the control group ingested a similar volume of a non-caloric, commercially available diet soda. After 28 days of ingestion, participants who consumed the ED experienced statistically greater decreases in percent body fat (ED: 25.6 ± 1.4 to 25.4 ± 1.5 % vs Control: 25.1 ± 1.5 to 25.9 ± 1.5 %) and fat mass (ED: 18.9 ± 1.5 to 18.3 ± 1.5 kg vs. Control: 19.1 ± 1.3 to 18.4 ± 1.2 kg) when compared to the control group, albeit minimal. In addition, area under the curve values for free fatty acids were greater 28 days after ingesting the ED when compared to placebo values while no changes were observed in metabolic rate and glycerol values. Also worth noting, there were no differences between groups concerning blood and clinical safety markers. However, one participant in the ED group withdrew from the study because of reoccurring symptoms of gastrointestinal distress. A follow-up investigation from this research team performed a sex-based analysis revealing that women had higher free fatty acid area under the curve values when compared to men, however, males who ingested the ED lost significantly more body fat [219].

A study by Lockwood et al [225] examined the longer-term impacts of ED consumption (10 weeks exposure). This study lasted 10 weeks and randomly assigned 38 previously sedentary males to one of four groups: ED + exercise, ED + no exercise, placebo + exercise, and placebo + no exercise. Before and after 10 weeks of supplementation, participants were assessed for changes in body composition, cardiorespiratory fitness, strength, mood, and adverse events. ED consumption without exercise was not responsible for any changes in body composition, fitness, or strength. However, when ED consumption was combined with a weekly exercise program, greater improvements in body composition and peak oxygen uptake were observed when compared to exercise + placebo ingestion. The authors concluded the ED ingestion by itself did not exert any favorable outcomes related to body composition or performance, but when combined with a weekly exercise program, ED consumption may augment improvements in body composition and oxygen capacity as a result of the accumulative benefits from improvements in acute performance and exercise capacity. As highlighted earlier in this section, a key consideration for people interested in weight and fat loss outcomes is the energy content of the beverage consumed. Additionally, the authors did not observe any changes in clinical markers for hepatic, renal, cardiovascular, and immune function, in response to the energy drink.

## 16. Safety considerations

Caffeinated beverages are some of the most widely consumed drinks worldwide, with average daily caffeine intakes ranging between 160-215 mg∙day^−1^ [226,227]. Daily caffeine intake appears to be highest in adults between the ages of 50-64 and the 90^th^ percentile intake has been found to be 380 mg∙day^−1^ for all ages, with coffee representing the primary contributor of daily caffeine intake [227,228]. While commercially available and regularly consumed by millions of individuals annually, there continue to be concerns regarding the safety of both acute and long-term consumption of ED products, particularly when combined with alcohol. However, the majority of evidence in support of such claims are often based upon epidemiological cross-sectional study designs and correlational evidence, thereby limiting the ability to discern direct causality or differentiate between potential causality and reverse causality. Importantly, the total caffeine intake from all sources in the diet beyond just ED or ES alone should be considered when evaluating the potential health risks, as individuals may consume caffeine from a variety of sources throughout the day. Thus, if adverse events occur, a truer analysis of potential causality can be better assessed. For example, ED consumption has been associated with higher self-reported ratings of stress, anxiety, and depression-related symptoms with continued use [7,229,230]. However, individuals with such underlying mental health issues may be more prone to consume ED to cope with the symptoms, rather than ED consumption causing these issues directly. To address these limitations, Kaur et al. utilized a prospective longitudinal study design to assess changes in self-reported ratings of anxiety and stress in young adults [231]. The authors found that males, but not females who changed from being a non‐ED user to an ED user had an average increase in depression, anxiety, and stress scores of 6.09 (95% CI = 3.36, 8.81), 3.76 (95% CI = 1.82, 5.70), and 3.22 (95% CI = 0.47, 5.97), respectively. While it is acknowledged that certain ingredients commonly found in EDs (i.e. caffeine) may exacerbate underlying symptoms associated with stress and anxiety [232-234], the challenge then becomes identifying which of the ingredients, or the potential combination of ingredients, may be responsible for these changes. Furthermore, it is unknown as to why certain people may respond more favorably regarding the acute physiological and cognitive effects, while others may perceive these effects as negative. Regardless, these associations warrant further investigation to better understand the influence of ED consumption on mental health and issues pertaining to stress, anxiety, and depression.

The most commonly reported adverse effects from ED consumption include insomnia, stress, depressive mood, jitteriness/restlessness, and gastrointestinal upset [235]. In addition, several published case reports, original investigations, and reviews are available which indicate that acute ED ingestion may be linked to various cardiovascular adverse effects including disruptions in cardiac rhythms, vascular function, and hemodynamic responses in addition to other more benign adverse effects such as irritability, nausea, and jitteriness [235-238]. However, it is again not possible to infer direct causality from individual case reports, as they do not account for various confounding variables such as underlying medical conditions, medications, volume of ED consumed, and other co-ingested ingredients in addition to not being generalizable to a larger population. Rather, it is important to review randomized controlled trials that have controlled for confounding variables and evaluated the short-term effects of ED consumption under controlled conditions, using valid instruments and study procedures. To address this, Shaw et al. [239] utilized a non-caffeine control to assess acute effects of ED consumption. The authors observed significant differences in peripheral (ED 1: 15.9 ± 5.0 mm Hg; ED 2: 14.4 ± 4.8 mm Hg; Placebo: 9.8 ± 4.8 mm Hg) and central systolic blood pressure (ED 1: 11.1 ± 4.7 mm Hg; ED 2: 10.1 ± 4.8 mm Hg; Placebo: 6.5 ± 3.5 mm Hg) and peripheral (ED 1: 9.6 ± 4.1 mm Hg; ED 2: 9.6 ± 4.9 mm Hg; Placebo: 6.1 ± 3.8 mm Hg) and central diastolic blood pressure (ED 1: 9.9 ± 4.2 mm Hg; ED 2: 9.8 ± 5.1 mm Hg; Placebo: 6.7 ± 3.5 mm Hg) in young healthy adults following ingestion of two different EDs (containing 304–320 mg of caffeine per 32-fl oz., taurine, glucuronolactone, and vitamins along with other proprietary ingredients) compared to a placebo control. Similar findings have been observed in previous investigations, in which analogous cardiovascular responses, hemodynamic perturbations, electromyographic, and electrolyte disturbances have been reported following acute ED ingestion [240-243]. Franks and colleagues [244] studied nine healthy, young (27.7 ± 5.0 yrs.) adults (5 F, 4 M) and had them consume in a crossover fashion either four doses of an ED or a caffeinated placebo in a single day. Each of the four doses were separated by approximately four hours and they were assessed for changes in heart and blood pressures. When the ED was consumed, significantly higher systolic (123.2 vs. 117.4 mm Hg) and diastolic blood pressures (73.6 vs. 68.2 mm Hg) as well as higher mean arterial pressures (90.1 vs. 84.8 mm Hg), were recorded over a 24-hour period. Furthermore, a recent meta-analysis [245] concluded that acute ingestion of EDs leads to significant increases in resting systolic and diastolic blood pressure (4.44 mm Hg [95% CI = 2.71 to 6.17; Cochrane Q P = 0.001] and 2.73 mm Hg [95% CI = 1.52 to 3.95; Cochrane Q P = 0.050)], respectively), however other studies have failed to observed significant hemodynamic or ECG changes when compared to a control, with only mild to no elevations in heart rate and blood pressure reported [221,246].

As mentioned previously, a challenge when drawing comparisons across the literature is the variability of ED ingredient profiles [2], and the volume of fluid (and caffeine content) consumed in each investigation. As such, it is difficult to identify the primary ingredients responsible for any acute adverse cardiovascular responses. It is unlikely the caffeine content alone is responsible for the previously mentioned adverse events that may be associated with ED consumption as the average caffeine content in EDs is ~159 mg per serving. However, it is unknown which, if at all, other ingredients may synergistically influence the risk of adverse effects. Additionally, habitual intake, total caffeine consumption throughout the day, and genetic profile may all influence the risk of adverse effects. While the amount of caffeine commonly found in ED’s is higher than most soft drinks, it is only slightly above the caffeine content for an 12 oz. serving of brewed coffee (~150 mg), which has not been found to be associated with elevated risks of adverse effects or abnormal electrocardiographic or hemodynamic responses [247]. Moreover, the FDA within the United States has concluded that caffeine in soft drinks is generally recognized as safe (GRAS) in the amount of up to 0.02% [248] and that up to 400 mg/day is not dangerous in healthy subjects, with a low risk of adverse events or cardiovascular complications. Certain medications and how fast individuals metabolize caffeine can make people more sensitive to caffeine and women that are pregnant, trying to become pregnant, or breastfeeding and therefore should avoid caffeine [18]. However, the combination of other ingredients, including but not limited to, other stimulants, vitamins, electrolytes, herbal extracts, or other botanicals may synergistically elevate risk of adverse events when consumed together. Additionally, ED are often flavored and served chilled, both of which make them easier to consume much quicker than a hot beverage (i.e. coffee). Further, ES are much smaller in total fluid volume, and intended to be consumed in a matter of seconds. Therefore, people may be more susceptible to feeling the effects of the ED quicker, as they likely are consuming the beverages quicker, which could result in faster absorption rates of key active ingredients. However, it is difficult to discern direct causality in such instances as each ED has a proprietary ingredient profile, and therefore each drink may confer varying risks of adverse effects, if any at all. To address this limitation, Fletcher et al. [249] utilized a unique study design to assess the acute effects of ED consumption on ECG and hemodynamic parameters. The authors found higher corrected QT intervals with elevated systolic blood pressure following consumption of an ED compared to a caffeine-matched control beverage in young healthy adults, which is a noteworthy side effect as elevated or prolonged QTc intervals is a known risk factor for cardiovascular events [250]. Additionally, systolic blood pressure was raised in both conditions initially, however systolic blood pressure was significantly higher at six hours post ingestion of the ED when compared with the caffeine matched control (4.72 ± 4.67 mm Hg vs. 0.83 ± 6.09 mm Hg, respectively; p = 0.01), suggesting some of the additional ingredients in the ED may elicit hemodynamic actions. It is unknown if blood pressure values were still within normal ranges, as the raw values were not presented. No differences were observed between groups at any time point for heart rate, diastolic blood pressure, central systolic blood pressure, and central diastolic blood pressure. Similar findings were reported by Phan et al. [251], who found that consumption of a caffeinated ES led to acute increases in peripheral and central systolic blood pressures compared to a non-caffeinated energy shot control drink. Kurtz et al. [252] confirmed these findings by similarly reporting significant changes in systolic and diastolic blood pressure following consumption of a caffeinated ES compared to non-caffeinated ES control. Interestingly, the authors reported similar heart rate responses, adverse effects, and energy levels in participants across both conditions. Overall, these findings collectively indicate that it is likely that caffeine is a primary contributor to the arrhythmogenic responses commonly observed. However, it is unknown how the combination of the additional ingredients found in ED/ES, in conjunction with the caffeine, further influences arrhythmogenic responses. As such, more research needs to be completed as it is not clear how long hemodynamic values stay elevated (if at all) after regularly ED consumption. Finally, one must also consider acute changes of these magnitudes may lack clinical relevance, particularly if elevated for brief periods of time, unless multiple beverages are consumed throughout the day.

The long-term implications of these acute cardiovascular responses after consumption of multiple ED beverages for an extended period of time (i.e. months or years) are currently unknown, and therefore such habits are currently not recommended. Moreover, the majority of the literature on this topic has focused on young healthy adults. As such, the implications of these acute cardiovascular responses among populations with comorbidities, specifically cardiovascular or metabolic disease, are also currently unknown. It is therefore recommended that individuals with diabetes, cardiovascular disease (specifically hypertension), and neurological disorders consult their primary care physician regarding the potential risks of ED/ES consumption.

## 17. Final summary and conclusions

The following 13 points constitute the Position Statement of the Society. They have been approved by the Research Committee of the Society:

1. Energy drinks (ED) commonly contain caffeine, taurine, ginseng, guarana, carnitine, choline, B vitamins (vitamins B1, B2, B3, B5, B6, B9, and B12), vitamin C, vitamin A (beta carotene), vitamin D, electrolytes (sodium, potassium, magnesium, and calcium), sugars (nutritive and non-nutritive sweeteners), tyrosine, and L-theanine, with prevalence for each ingredient ranging from 1.3 to 100%.
2. Energy drinks can enhance acute aerobic exercise performance, largely influenced by the amount of caffeine (> 200 mg or >3 mg∙kg bodyweight [BW^−1^]) in the beverage.
3. Although ED and ES contain several nutrients that are purported to affect mental and/or physical performance, the primary ergogenic nutrients in most ED and ES based on scientific evidence appear to be caffeine and/or the carbohydrate provision.
4. The ergogenic value of caffeine on mental and physical performance has been well-established, but the potential additive benefits of other nutrients contained in ED and ES remains to be determined.
5. Consuming ED and ES 10-60 minutes before exercise can improve mental focus, alertness, anaerobic performance, and/or endurance performance with doses >3 mg∙kg BW^−1^.
6. Consuming ED and ES containing at least 3 mg∙kg BW^−1^ caffeine is most likely to benefit maximal lower-body power production.
7. Consuming ED and ES can improve endurance, repeat sprint performance, and sport-specific tasks in the context of team sports.
8. Many ED and ES contain numerous ingredients that either have not been studied or evaluated in combination with other nutrients contained in the ED or ES. For this reason, these products need to be studied to demonstrate efficacy of single- and multi-nutrient formulations for physical and cognitive performance as well as for safety.
9. Limited evidence is available to suggest that consumption of low-calorie ED and ES during training and/or weight loss trials may provide ergogenic benefit and/or promote additional weight control, potentially through enhanced training capacity. However, ingestion of higher calorie ED may promote weight gain if the energy intake from consumption of ED is not carefully considered as part of the total daily energy intake.
10. Individuals should consider the impact of regular coingestion of high glycemic index carbohydrates from ED and ES on metabolic health, blood glucose, and insulin levels.
11. Adolescents (aged 12 through 18) should exercise caution and seek parental guidance when considering the consumption of ED and ES, particularly in excessive amounts (e.g. >400 mg), as limited evidence is available regarding the safety of these products among this population. Additionally, ED and ES are not recommended for children (aged 2-12), those who are pregnant, trying to become pregnant, or breastfeeding and those who are sensitive to caffeine.
12. Diabetics and individuals with preexisting cardiovascular, metabolic, hepatorenal, and/or neurologic disease who are taking medications that may be affected by high glycemic load foods, caffeine, and/or other stimulants should exercise caution and consult with their physician prior to consuming ED and ES.
13. The decision to consume ED or ES should be based upon the beverage’s content of carbohydrate, caffeine, and other nutrients and a thorough understanding of the potential side effects. Indiscriminate use of ED or ES, especially if multiple servings per day are consumed or when consumed with other caffeinated beverages and/or foods, may lead to adverse effects.
